# Next Generation Sequencing-Based Analysis of Repetitive DNA in the Model Dioceous Plant *Silene latifolia*

**DOI:** 10.1371/journal.pone.0027335

**Published:** 2011-11-09

**Authors:** Jiří Macas, Eduard Kejnovský, Pavel Neumann, Petr Novák, Andrea Koblížková, Boris Vyskot

**Affiliations:** 1 Biology Centre of the Academy of Sciences of the Czech Republic, Institute of Plant Molecular Biology, České Budějovice, Czech Republic; 2 Institute of Biophysics, Brno, Czech Republic; Auburn University, United States of America

## Abstract

**Background:**

*Silene latifolia* is a dioceous plant with well distinguished X and Y chromosomes that is used as a model to study sex determination and sex chromosome evolution in plants. However, efficient utilization of this species has been hampered by the lack of large-scale sequencing resources and detailed analysis of its genome composition, especially with respect to repetitive DNA, which makes up the majority of the genome.

**Methodology/Principal Findings:**

We performed low-pass 454 sequencing followed by similarity-based clustering of 454 reads in order to identify and characterize sequences of all major groups of *S. latifolia* repeats. Illumina sequencing data from male and female genomes were also generated and employed to quantify the genomic proportions of individual repeat families. The majority of identified repeats belonged to LTR-retrotransposons, constituting about 50% of genomic DNA, with Ty3/gypsy elements being more frequent than Ty1/copia. While there were differences between the male and female genome in the abundance of several repeat families, their overall repeat composition was highly similar. Specific localization patterns on sex chromosomes were found for several satellite repeats using in situ hybridization with probes based on *k*-mer frequency analysis of Illumina sequencing data.

**Conclusions/Significance:**

This study provides comprehensive information about the sequence composition and abundance of repeats representing over 60% of the *S. latifolia* genome. The results revealed generally low divergence in repeat composition between the sex chromosomes, which is consistent with their relatively recent origin. In addition, the study generated various data resources that are available for future exploration of the *S. latifolia* genome.

## Introduction

*Silene latifolia* (family Caryophyllaceae) is a dioceous plant possessing heteromorphic sex chromosomes and a sex determination system similar to the human type, with heterogametic male (XY) and homogametic female (XX) individuals [1]. It has an intermediate genome size, which differs between males (5.85 Gb/2C) and females (5.73 Gb/2C) due to the unequal size of the X and Y chromosomes [2], [3]. A substantial fraction of the *S. latifolia* genome is composed of repetitive DNA and this type of genomic sequence is also supposed to be involved in the differentiation of its sex chromosomes [4]. Indeed, several repeats with specific localization patterns have been identified on the sex chromosomes, including satellite [5] and simple sequence repeats [6] and plastid DNA [7]. To date, the most complex analysis of repetitive DNA was performed by sequencing 379 clones from a *S. latifolia* short-insert genomic library and subsequent FISH analysis of selected clones. In addition to identification of the chromosome Y-specific tandem repeat STAR-Y, this study revealed depletion of Ogre-like retrotransposon sequences from the non-recombining part of the Y chromosome [8]. Despite this progress, there remains a lack of detailed sequence and quantitative information about the global repeat composition of the *S. latifolia* genome, similar to that available for extensively sequenced model species.

The introduction of next generation sequencing (NGS) technologies, based on the fast and cost-efficient parallel processing of millions of templates, has revolutionized many areas of the current life sciences [9]. The power of NGS, which can generate up to gigabases of sequence data in a single run, has presented new opportunities for the investigation of highly complex populations of repetitive elements in plant genomes [10]-[12]. The procedure is cloning-free, thus avoiding the potential bias caused by difficulties in propagating some repeat types in bacteria [13], and provides random sequence sampling across the genome. In principle, repeat detection is then based on evaluating the frequencies of identical or similar sequence reads, which increase as the genomic copy numbers of corresponding repetitive elements increase. An example of the straightforward application of this principle is the detection of repetitive elements in sequences of genomic clones based on the numbers of similarity hits to databases of low-pass whole genome sequencing data [12], [14]. In order to achieve *de novo* identification of repetitive elements without the need for reference genomic sequences, we introduced a similarity-based read clustering approach that detects repetitive sequences as groups of frequently overlapping sequence reads [10]. The clustering procedure has recently been improved by employing graph-based methods, transforming read similarities revealed by all-to-all comparison to a virtual graph, where the reads are represented by nodes and their mutual similarities by edges connecting the nodes. Graph topology is then examined to identify and further characterize communities of densely connecting nodes representing families of repetitive sequences [15]. This approach proved to be efficient for identification of repeats in several plant species including pea, soybean, tobacco and potato [15]–[17].

In this work, we aimed to carry out a global analysis of the repetitive fraction of the *S. latifolia* genome using next generation sequencing combined with the bioinformatics approaches described above. We used relatively long reads generated by 454 sequencing to perform clustering-based identification and reconstruction of all major types of genomic repeats. This analysis resulted in partitioning the sequencing data into groups (clusters) of reads, each of which contained sequences derived from a single family of repetitive element. Then, we estimated the genomic abundance of these repeat families using data obtained by sequencing male and female genomes on the Illumina platform. We employed 454 read clustering data as a reference for repeat identification in the Illumina data, as the short length of Illumina reads did not allow analysis of their clustering. On the other hand, Illumina sequencing was chosen for repeat quantification due to substantially larger numbers of generated reads, providing better genome sampling compared to the 454 sequencing. In addition, this platform is more reliable for sequencing and quantification of satellite sequences, and thus the Illumina data were also used for detailed characterization of the satellite DNA, which represents one of the most dynamic components of plant genomes.

## Results

### 454 sequencing, repeat identification and classification

**Figure 1:**
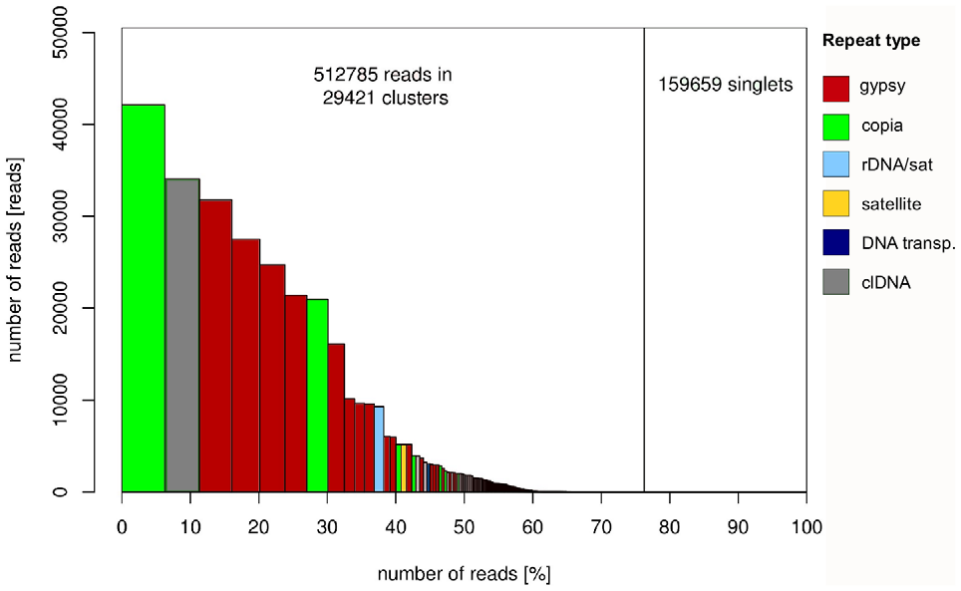
Size distribution and repeat composition of clusters generated by similarity-based partitioning of *S. latifolia* 454 reads. Bars on the histogram represent individual clusters, bar sizes correspond to number of reads in the clusters and colors to the type of repetitive sequences. The cumulative proportion of clusters in the genome is shown along the X-axis.

One sequencing run on the 454 GS FLX platform was performed for each male and female genomic DNA, yielding 250,507 and 421,937 quality-filtered reads, respectively. The average read length was 257 nucleotides for the male and 231 nucleotides for the female sample. The two read sets were combined for further analysis, providing a total of 161.7 Mb of sequence data, corresponding to about 5.6% of the haploid genome size. Similarity-based clustering of the reads resulted in 29,421 clusters containing from 2 up to 42,110 reads. The clusters included 76% of all analyzed reads, with 50% of the reads being assigned to the 46 largest clusters representing the most abundant repetitive elements in the genome (Fig. 1). Given the low genome coverage of the sequencing (0.056x), which makes the clustering of reads derived from single-copy sequences unlikely, the observed proportion of clustered reads revealed a high content of repetitive DNA in the *S. latifolia* genome.

In order to classify repeats in the individual clusters, contigs assembled from the reads as well as individual read sequences were subjected to similarity searches against various sequence databases. They were also investigated for the presence of specific structural features like tandem subrepeats or potential retroelement long terminal repeats (LTRs). In addition, the shapes of the cluster graph layouts (Fig. S1) were examined because they can be used to distinguish basic repeat types [15]. Taken together, these analyses allowed us to classify repeat types in most of the large clusters (Fig. S1) and revealed the presence of diverse groups of repeats. Although the largest cluster contained sequences of Ty1/copia LTR-retrotransposons (Angela family), Ty3/gypsy were found to be the prevailing type of repeats in the *S. latifolia* genome (Fig. 1). All major lineages of Ty3/gypsy elements known from plant genomes were detected and distinguished based on phylogenetic analysis of their reverse transcriptase (RT) coding domains, while there were only two (Angela and Maximus, [18]) identified based on Ty1/copia RT domains (Fig. S2).

**Figure 2:**
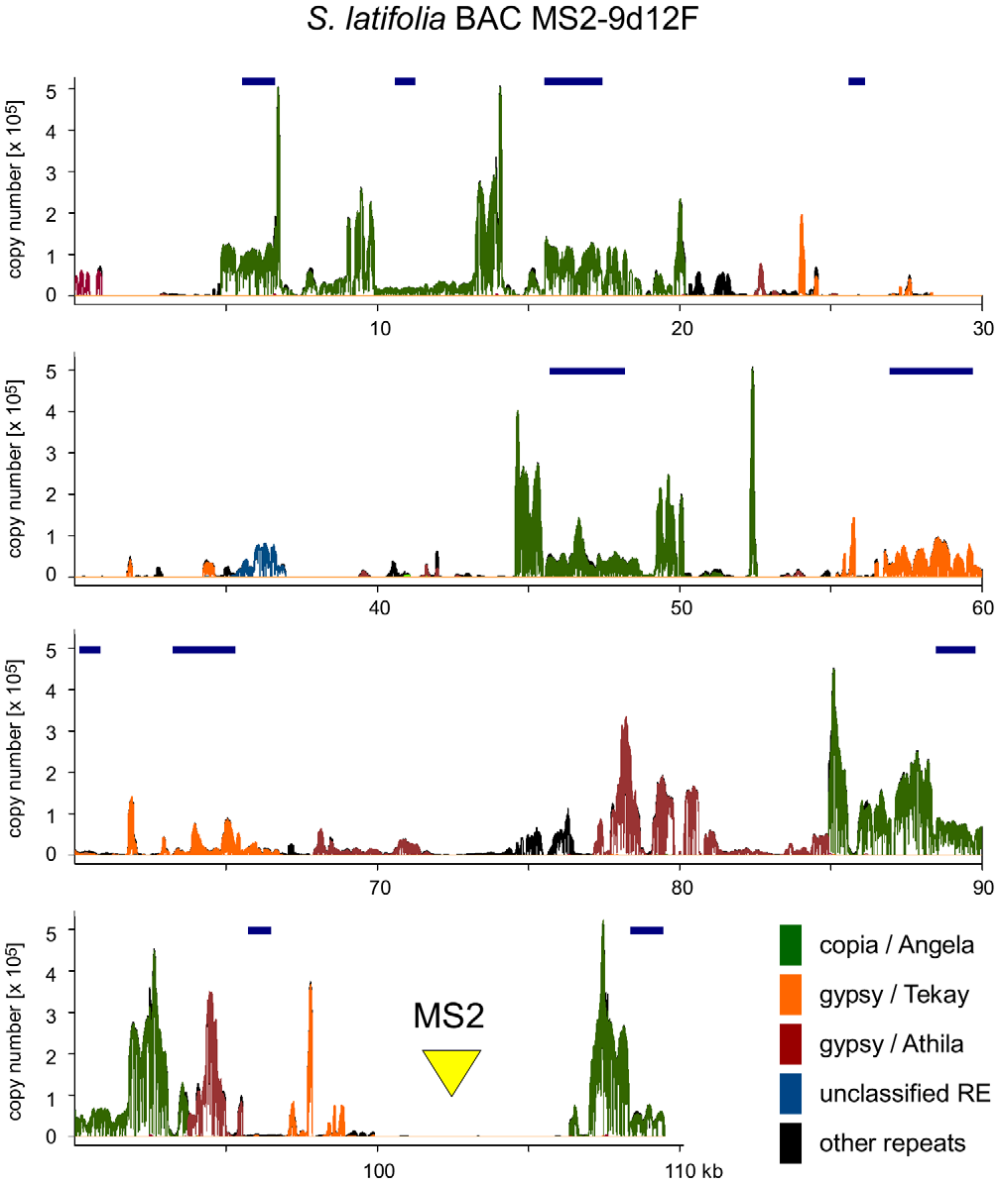
Repeat identification in the clone of *S. latifolia* genomic DNA using similarity search against repeat-specific 454 read databases. Sequence of the chromosome Y-derived BAC clone MS2–9d12F (109.5 kb, GenBank accession AB257588) containing genomic regions surrounding molecular marker MS2 [19] was searched for similarities to a set of databases compiled from the 454 reads assigned to individual repeat families. The search was performed using the PROFREP server employing blastn program with an e-value cutoff of 1e^−15^. Numbers of similarity hits along the sequence were converted into copy numbers by their normalization to the genome sequencing coverage of 454 sequencing (0.056x). The plot colors correspond to different families of repetitive DNA and the position of the MS2 sequence is marked by a yellow triangle. Bars above the plot show the positions of putative retrotransposon coding domains detected by Ishii et al. [19].

Since the output of these analyses as well as the contig sequences and assembly files provide valuable material for detailed investigation of the structure and variability of individual repeat families, we have made them available in full for future studies (see Electronic resources). An additional tool for genome investigation in *Silene* was generated by merging reads from clusters containing the same repeat types/families, resulting in repeat-specific databases that can be used in various types of sequence similarity searches. The advantage of using such databases instead of a few selected consensus sequences is that they capture the full range of the repeat sequence variation and thus provide better sensitivity in the detection of less conserved repeat variants. A similarity search of these databases using the sequence of the chromosome Y-derived BAC clone MS2–9d12F [19] as a query demonstrated their utility in repeat detection and annotation in genomic clones (Fig. 2). The results were consistent with the published annotation of this BAC sequence, showing the absence of repeats in the region containing the Y-specific marker MS2 and the presence of retrotransposons in most of the regions previously found to have similarity to gag-pol proteins [19]. However, our approach provided additional information about retrotransposon types, their mutual arrangement and the presence of other repetitive sequences (Fig. 2).

### Repeat quantification using Illumina sequencing data

Illumina sequencing was performed with separate samples of male and female total genomic DNAs as templates, using a protocol generating 36-nucleotide reads. The reads were trimmed to 30-nucleotide high-quality sequence tags, resulting in 5.2 million tags for the male sample and 8.9 million tags for the female sample. Five million tags were randomly sampled from each dataset in order to use the same amount of sequence data for comparative analysis of the male and female sample. The tags were then assigned to the previously identified repeats based on their sequence similarities to 454 reads within the repeat clusters. The similarity threshold for the tag assignment allowed a maximum of two mismatches and each tag was assigned to a maximum of one cluster based on its best similarity. The numbers of tags mapped to individual clusters were then used to quantify the proportions of the corresponding repeats in *S. latifolia*, and also to compare these proportions between the male and female genomes.

LTR-retrotransposons were found to be the dominant group of *S. latifolia* repeats, making up about 50% of the genome (Table 1). The identified Ty3/gypsy elements exceeded Ty1/copia by about 2.7-fold in terms of their proportion in the genome, with the Ogre family (Tat/Ogre lineage) and Athila lineage being the most abundant. The Angela lineage of Ty1/copia elements accounted for a similar proportion to the Ogre and Athila elements; however, other Ty1/copia lineages were much less frequent. Other repeats were found to make up considerably smaller genome proportions, including non-LTR retrotransposons represented by LINE elements and two superfamilies of DNA transposons. Over 3% of the genomic sequences comprised four families of satellite repeats, with the most abundant being the centromeric satellite STAR-C (1.9%).

**Table 1:** Repeat composition of *S. latifolia* genome estimated from Illumina sequencing data.

Repeat			Genome proportion [%] (1)	
Type	Lineage	Clade/Family	Male	Female
***Retroelements***			*(49.7)*	*(51.4)*
Ty3/gypsy			*(35.7)*	*(37.5)*
	Tat/Ogre			
		Ogre	11.94	13.24
		Tat	2.38	2.51
	Athila		11.95	12.22
	chromovirus			
		Tekay	6.90	6.99
		CRM	0.26	0.26
		other	0.34	0.35
Ty1/copia			*(13.7)*	*(13.7)*
	Angela		11.75	11.83
	Maximus		1.65	1.64
	other		0.28	0.27
TRIM			0.04	0.04
LINE			0.23	0.23
***DNA transposons***			*(1.45)*	*(1.44)*
CACTA			1.25	1.25
Mutator			0.20	0.19
***Satellite repeats***			*(3.09)*	*(3.19)*
		STAR-C	1.92	1.93
		X43.1^(2)^	0.99	1.11
		15Ssp	0.09	0.08
		TRAYC-like	0.09	0.08
***rDNA***				
45S rDNA^(3)^			0.31	0.44
5S rDNA			0.03	0.04
***unclassified***			6.89	6.72
**TOTAL**			**61.4**	**63.3**

^(1)^Genome proportions summarized for repeat types are given in parentheses.

^(2)^ Including X43.1 repeats located in rDNA spacer.

^(3)^ Excluding X43.1 repeats.

**Figure 3:**
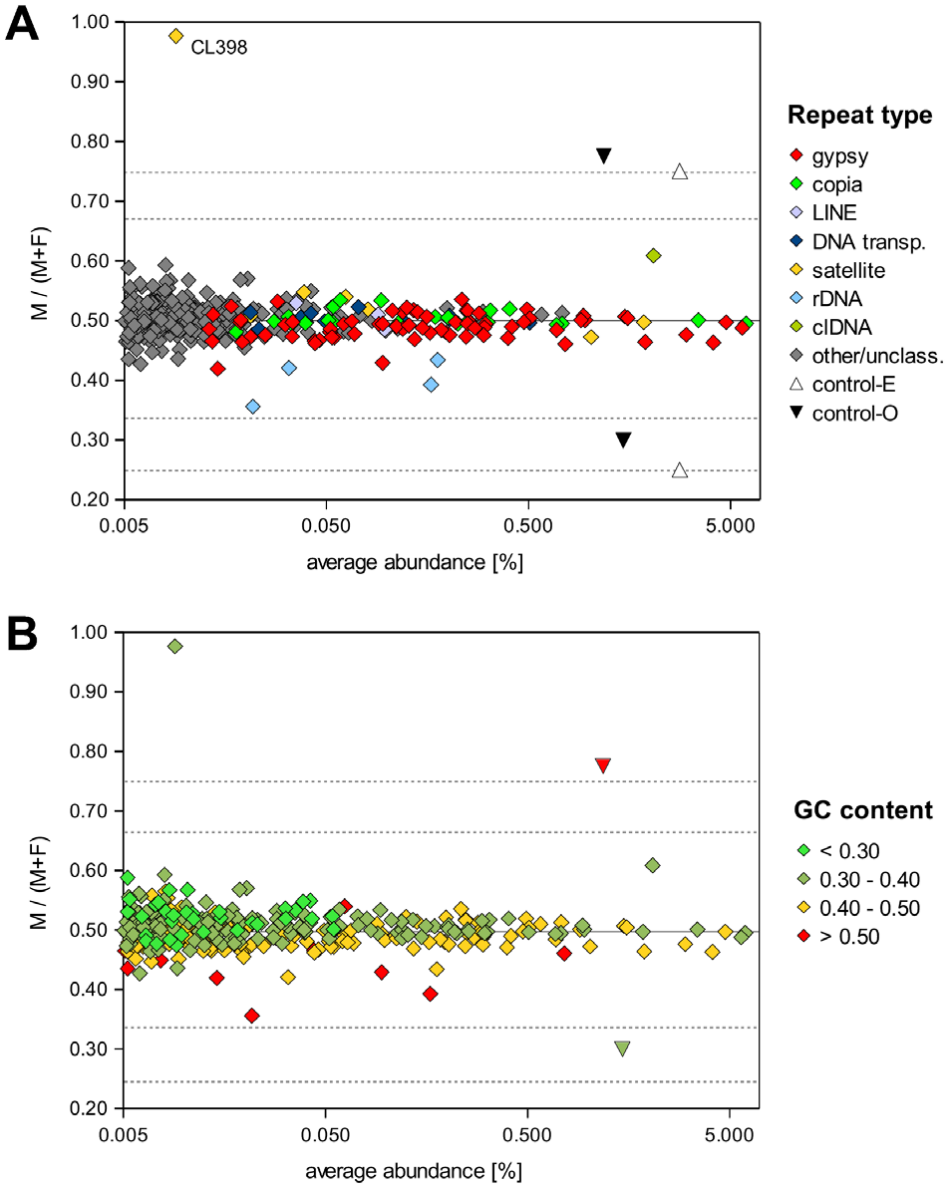
Comparison of genomic proportions of individual repeat families in male and female genomes. Symbols on the plot representing individual clusters are color-coded according to the repeat type (**A**) or proportion of GC in their sequences (**B**). The position along the X-axis corresponds to the average proportion of the repeat in male and female genomes, while its position along the Y-axis is determined by its relative abundance in male (M) and female (F) genomes. This is expressed as the repeat proportion in the male divided by the sum of its proportions in the male and female genomes, resulting in the value of 0.5 for sequences with the same proportions in male and female genomes (marked by a solid line on the graph). The dashed lines mark two- and three-fold enrichment of a sequence in the male (corresponding to the values of 0.67 and 0.75, respectively) and female (0.33 and 0.25, respectively) genome. The positions of the sequence quantification controls are marked by triangles, showing their expected (open symbols) and observed (black symbols) values.

Since the *S. latifolia* male and female genomes differ only by the constitution of their sex chromosomes, the eventual differences found in repeat composition between these genomes should reveal sequences with differential distribution on the X and Y chromosomes. The repeat quantification based on Illumina data indicated that the abundance of most of the repeat families is similar in males and females (Fig. 3 and Table 1). In order to verify the sensitivity and accuracy of the quantification process, the sequenced samples were spiked-in with known amounts of two control sequences represented by lambda and T4 phage DNAs. The controls were added to the genomic DNA samples before sequencing in amounts corresponding to a total of 3% of the sample DNA; however, the ratio of lambda to T4 DNA was different, being 3∶1 in the male and 1∶3 in the female genome sample. These three-fold differences in control DNA amounts were readily distinguished in the quantification data; the observed proportions of lambda/T4 DNA were 1.75%/0.92% (expected values were 2.1%/0.7%) in the male and 0.51%/2.17% (0.7%/2.1% expected) in the female sample. Out of the 370 largest clusters of repeats with genome proportions of at least 0.005%, only one (CL398) was found to differ more than the controls between the male and female sequence data (Fig. 3A). The repeat represented by CL398 was enriched over 42-fold in the male compared to the female sample and was found to contain tandem repeats with high similarity to the chromosome Y-specific variant of the STAR-C satellite (STAR-Y, [8], [20]). The predominant localization on the Y chromosome was also revealed using FISH with an oligonucleotide probe designed to specifically distinguish CL398 sequences. All other observed differences in repeat proportions were significantly smaller and fell below the two-fold range (Fig. 3A). There was a slight enrichment of retrotransposon Ogre sequences in the female DNA (Table 1) which is consistent with the observed absence of these elements from the non-recombining part of the Y chromosome [8]. Chloroplast DNA sequences (clDNA) were enriched in the male sequence data (Fig. 3A) but judging from their high sample proportions (1.6–2.5%) they probably originated from plastid sequences unequally contaminating nuclear DNA preparations rather than from relatively rare insertions of clDNA into nuclear genome [7]. An unexpected finding was about 1.4-fold higher proportion of ribosomal DNA (rDNA) sequences in the female sample (Fig. 3A), because all rDNA loci are known to occur on autosomes [21] and thus this result cannot be explained by their accumulation on the X chromosome. Indeed, real-time PCR quantification of two rDNA regions including parts of the 18S and 25S genes in the DNA samples used as the sequencing templates did not confirm their different copy numbers (data not shown), indicating that they were generated during sequencing. Further investigation of the Illumina sequencing data revealed that the enrichment in the female sample was proportional to the elevated GC content of rDNA sequences (Fig. S3). Since the sequencing quality of this sample was better than in the case of male DNA (1.7-fold more high quality tags were obtained) and it has been reported that GC-rich templates are prone to frequent sequencing errors [22], it is likely that differences in the efficiency of sequencing these templates caused the observed bias in rDNA representation. It should be noted, however, that there were only a few repeats with a GC content as high as in the rDNA (Fig. 3B) and therefore this bias did not significantly affect the output of quantification analysis.

### Satellite repeats

**Figure 4:**
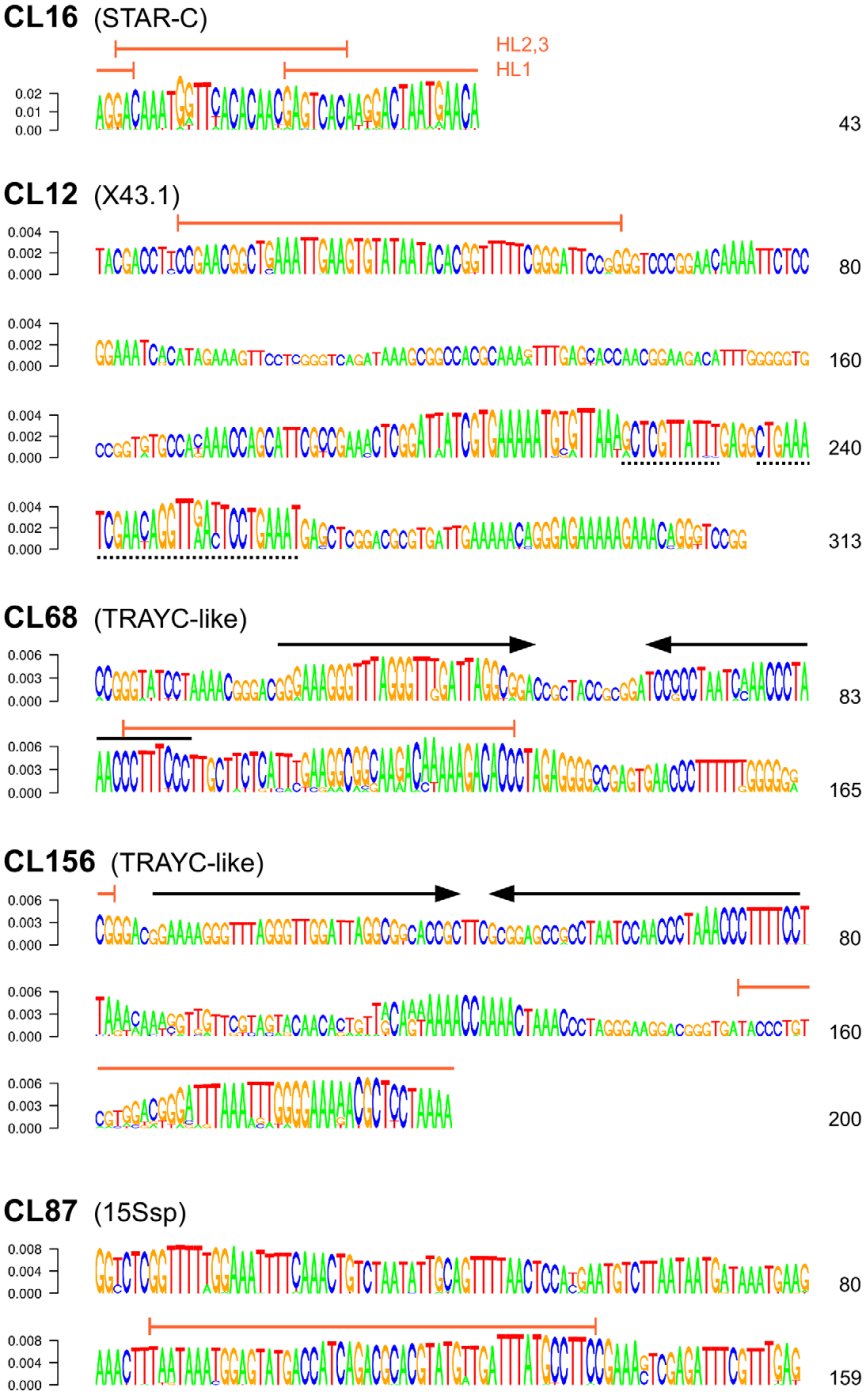
Consensus sequences of *S. latifolia* satellite repeats reconstructed from the most frequent *k*-mers. *K*-mer frequencies for each satellite were calculated from Illumina sequencing data and used to reconstruct their most conserved fragments. These fragments were merged to create full-length monomers as depicted in Fig. S4. The resulting consensus sequences are displayed as sequence logos where the height of the letters reflects the frequencies of the corresponding *k*-mers, and major sequence variants are displayed along with the prevailing nucleotides [23]. The sequence regions used to design the FISH probes are marked with orange lines and the palindrome sequences common to the CL68 and CL156 repeats are marked with black arrows. The dashed underlines in the CL12 sequence show the positions of two regions that are duplicated in some of the X43.1 monomers.

There were four families of satellite DNA identified in the *S. latifolia* NGS data (Table 1), displaying various extents of similarity to previously reported tandem repeats. The large volumes of available sequence data allowed us to perform, for the first time, a global analysis of their sequences, including detection of the most conserved regions, reconstruction of consensus sequences of repeat monomers and identification of major sequence variants (subfamilies). This was achieved by evaluating the frequencies of *k*-mers (oligomers of length *k*) in the Illumina data from clusters containing tandem repeats and using this information to reconstruct the most frequent sequence variants [23]. The output of this analysis (Fig. 4 and Fig. S4) was used to design hybridization probes and investigate their localization on *S. latifolia* chromosomes using FISH.

**Figure 5:**
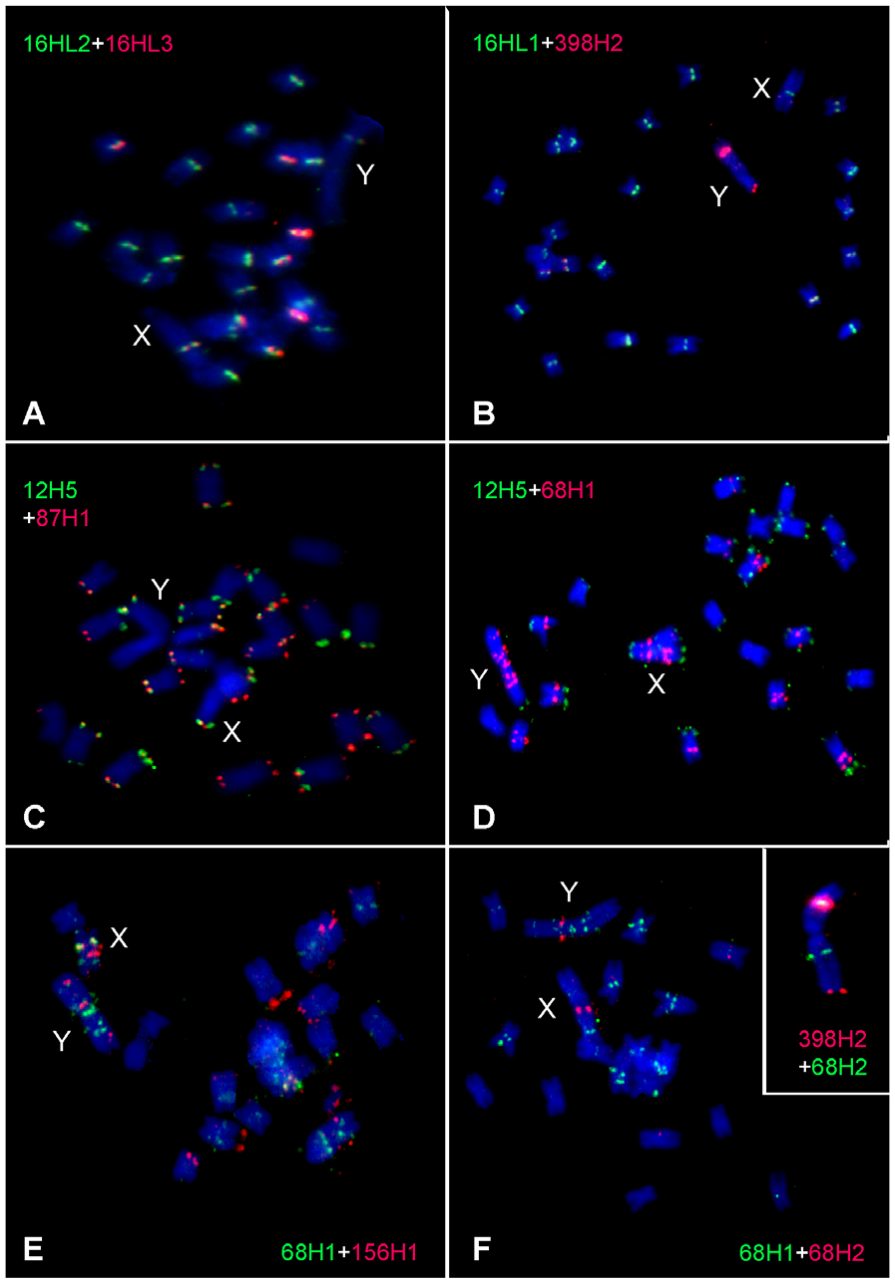
Localization of satellite repeats on metaphase chromosomes of *S. latifolia*. FISH experiments were performed simultaneously with two probes labeled by different fluorochromes (red or green, as indicated) in order to investigate the co-localization of sequence variants of the STAR repeats (**A–B**), 15Ssp and X43.1 (**C**) and TRAYC-like repeats (**D–F**). (**A**) Co-localization of the CL16/STAR-C repeat consensus (16HL2) and its sequence variant (16HL3). (**B**) The probes for a different region of the CL16/STAR-C consensus (16HL1) and for the chromosome Y-enriched subfamily CL398/STAR-Y (398H2). (**C**) Consensus probes for CL87/15Ssp (87H1) and X43.1 (12H5) satellites. Co-localization of the CL68/TRAYC-like consensus (68H1) with X43.1 (12H5) (**D**), CL156 (156H1) (**E**) and with the CL68 subfamily (68H2) (**F**). The inset in (F) is an example of the Y chromosome hybridized to 68H2 and 398H2, showing the localization of their interstitial signals on different chromosome arms. The chromosomes were counterstained with DAPI (blue). Sex chromosomes are indicated with X and Y. The positions of probes within repeat monomers are shown on Fig. 4 and their sequences are provided in Table 2.

The most abundant satellite identified as cluster CL16 corresponded to the centromeric repeat STAR-C described by Cermak et al. [8]. This satellite makes up about 1.9% of the genome, corresponding to 1.6×10^6^ copies of its 43 bp monomers. The monomer sequences were highly homogenized, with only a few point mutations identified among the most frequent *k*-mers (Fig. 4). However, the probe 16HL3 specifically targeting these mutations produced FISH signals that differed in intensity from the consensus probe (16HL2, Table 2), indicating their unequal amounts in the centromeres of some chromosomes. In addition, they also differed with respect to labeling the Y chromosome, where only 16HL2 produced signal within the q-arm (Fig. 5A). This locus corresponded to the major signal produced by the related sequence of the chromosome Y-enriched repeat CL398/STAR-Y (Fig. 5B).

**Table 2:** Oligonucleotide probes used in FISH experiments.

Name	Sequence (5'->3')	Labeling
12H5	CCGAACGGCTGAAATTGAAGTGTATAATACACGGTTTTTCGGGATTCCGG	5'Cy3 or 5'FAM
16HL1	GAGTCACAA+GG+ACTAA+TGAACAAGGA (1)	3'FAM
16HL2	GACAAATG+GTTCA+CACAA+CGAGTCAC (1)	5'TYE563or 3'FAM
16HL3	GACAAATG+ATTCA+CACAA+TGAGTCAC (1)	5'TYE563
68H1	CTTTCCCTTGCTTCTCATTTGAAGGCGGCAAGACAAAAAGACAC	5'Cy3 or 5'FAM
68H2	CCTTTCCCTTGCTATTGTCATTCGAACACGAAAACCTAAAGACACC	5'Cy3 or 5'FAM
87H1	TAATAAATGGAGTATGACCATCAGACGCACGTATGTTGATTTATGCCTTC	5'Cy3 or 5'FAM
156H1	TACCCTGTCGTGGACGGGATTTAAATTTGGGGAAAAACGCTCCTAAAACG	5'Cy3
398H2	GTTCGCACAATGAGTCACAATGACAAGGACAAATGGTTCGCAC	5'Cy3

(1) Locked nuclei acid (LNA) oligonucleotides; the LNA bases are marked with “+”

**Figure 6:**
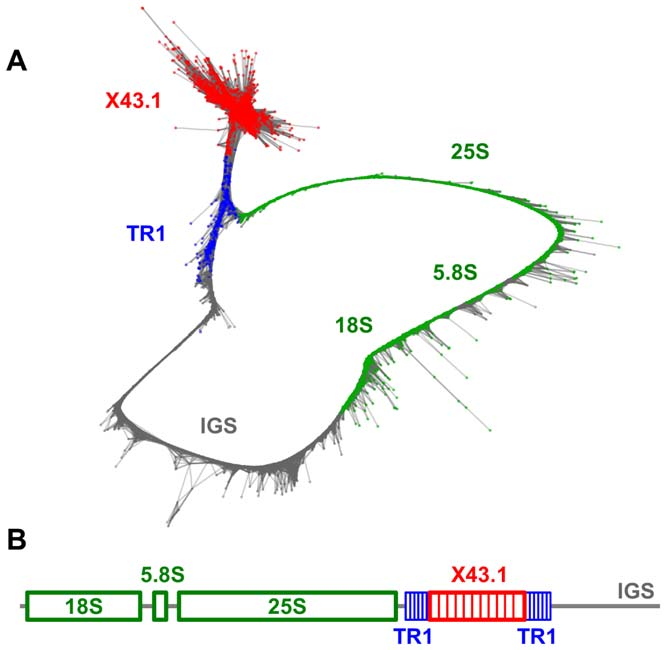
Graph representation and annotation of clusters containing rDNA genes and the satellite X43.1. (**A**) Graph layout displaying individual 454 reads as nodes and their similarities as connecting edges [15]. The circular shape of the graph reflects the tandem organization of multiple copies of the rDNA repeat units (reconstructed in **B**) in the genome. The graph nodes representing sequence reads from various parts of the rDNA unit are distinguished by colors. Sequences representing X43.1 repeats form due to their high copy number and tandem arrangement globular structure on the graph located at the 5' end of the intergenic spacer (IGS) and surrounded by another tandem repeat (TR1). The graph was created using merged sets of reads from clusters CL12 (containing rDNA genes, X43.1 repeats and part of the IGS) and CL59 (remaining part of the IGS).

Satellite X43.1 [24] was identified in cluster CL12, which, in addition to reads similar to X43.1, contained sequences derived from 45S rDNA repeats. Investigation of the cluster graph layout and assembled sequence reconstruction revealed that the cluster contained the almost complete rDNA repeat unit including 18S–5.8S–25S genes and the majority of the large intergenic spacer (IGS). The rest of the spacer was present in CL59, which was merged with CL12 for the purpose of complete rDNA unit reconstruction. The X43.1 repeats were found to be part of the IGS where they were surrounded by arrays of another tandem repeat, TR1 (Fig. 6). This finding explains the results of FISH experiments ([25] and data not shown), showing that some of the X43.1 signals co-localize with rDNA loci. Thus, it is likely that subtelomeric arrays of the X43.1 satellite found on most of the chromosomes originated by transposition and amplification of corresponding tandem subrepeats from the IGS, as proposed for several other plant satellites [26]. The X43.1 repeat makes up 1.0–1.1% of the genome (9.3–10.0×10^4^ copies/1C); its reconstructed consensus of 313 bp includes two regions that were duplicated in some monomers (Fig. 4), fully corresponding to the previously reported genomic sequences [8], [27], [28]. As this repeat has been subject to extensive characterization including FISH localization of its sequence variants [29], we did not investigate it further and used the probe derived from its consensus only in combination with other probes as a marker for discriminating X and Y chromosomes (it labels both arms of X but only one on the Y chromosome, Fig. 5C,D).

The cluster CL87 corresponded to the satellite 15Ssp [27], [28], with about 1.5×10^4^ copies of the 159 bp monomer occurring in the genome (Fig. 4). It was present in subtelomeric regions of all chromosomes, labeling both arms on most and co-localizing with the other subtelomeric satellite, X43.1. Similarly to X43.1, the 15Ssp signal on the Y chromosome was found on the q-arm only while it was localized on both arms of the X chromosome (Fig. 5C).

The most complex family of *S. latifolia* satellite repeats included sequences from the clusters CL68 and CL156. As these satellites shared partial similarity to the previously described GC-rich TRAYC repeat [5], we refer to them as TRAYC-like sequences. However, the similarity to the published TRAYC clone (GenBank accession DQ386857) was scattered across a region of only 73 bp, forming an imperfect palindrome in all TRAYC, CL68 and CL156 sequences. The palindrome was also the only similarity between the CL68 and CL156 families, which otherwise differed in their sequences as well as in monomer lengths, being 165 bp in CL68 and 200 bp in CL156 (Fig. 4). Based on our genome sequencing data it appears that the TRAYC clone is a very rare sequence variant because it could not be detected by the similarity search against the entire 454 and Illumina datasets (not shown). On the other hand, CL68 repeats were found to be relatively abundant, with an estimated 1.2×10^4^ and 1.0×10^4^ copies in the male and female genome, respectively, whereas CL156 was found to be present in males and females in similar quantities of about 3.1×10^3^ copies. The higher proportion of CL68 repeats compared to CL156 was supported by FISH results, which revealed CL68 signals on most autosomes and their strong accumulation on the sex chromosomes (Fig. 5D). On the contrary, the CL156 probe produced only a few signals on the sex chromosomes and several autosomes (Fig. 5E). In addition to sequence divergence between the families, sequence variation was also detected within the families, consisting of regions spanning up to tens of nucleotides that diverged considerably from the consensus (Fig. S4). FISH localization of the consensus CL68 probe (68H1) and one of these variants (68H2) revealed distinct patterns, showing that the 68H2 subfamily is mostly confined to one locus on each of the sex chromosomes and is absent from most genomic loci occupied by CL68 repeats (Fig. 5F). The dominant signal of the 68H2 probe on the Y chromosome was located on the p-arm while the strong signal of the CL398/STAR-Y probe was located on the q-arm (Fig. 5F).

## Discussion

In this work we performed the first comprehensive characterization of the repetitive fraction of the *S. latifolia* genome. The observed repeat content including the high proportion of LTR-retrotransposons is comparable to that found for other medium-sized plant genomes [10], [12], [30]. Although it is well established that retrotransposons represent one of the major forces causing extensive genome size variation in higher plants [30]–[33], it is yet to be determined what mechanisms lead to preferential amplification of the specific groups of retrotransposons observed in different taxa. In the *S. latifola* genome, Ty3/gypsy elements were almost three-fold more abundant than the Ty1/copia, with the Ogre elements being the largest repeat group in the genome. Ogres represent a phylogenetically and structurally distinct clade of the Tat lineage of Ty3/gypsy elements [34]. Due to their large size and amplification to high copy numbers they constitute up to 40% of nuclear DNA in some species [10], [31]. Using partial Ogre sequences as FISH probes, they were found to be dispersed on all *S. latifolia* chromosomes except for the majority of the Y chromosome, corresponding to its non-recombining region [8], [35]. Smaller proportions of Ogre-like repeats in male DNA compared to the female revealed in our Illumina data (Table 1 and Fig. 3) are consistent with these findings. Further evidence for Ogre depletion from the Y chromosome comes from the analysis of the chromosome Y-derived BAC clone MS2-9d12F, which completely lacks Ogre sequences although all other major *S. latifolia* retroelement families (Angela, Athila and Tekay) are well represented there (Fig. 2). However, the transposition or selective elimination mechanisms leading to this peculiar localization pattern of Ogre elements are yet to be elucidated.

In addition to revealing the sequence composition of repetitive DNA, the large amounts of sequencing data produced by NGS technologies make them attractive as a tool for repeat quantification. It has been shown that the genome proportions of various *P. sativum* repeats estimated using *in silico* analysis of 454 GS-20 sequencing data were in good agreement with their quantification using experimental methods, as the two estimates mostly differed by less than two-fold [10]. However, some features of the 454 sequencing procedure make it less favorable for quantification purposes. These include the occurrence of multiple identical reads originating as emulsion PCR artifacts [36] and less efficient sequencing of satellite repeats leading to underestimation of their abundance ([16] and our unpublished data). When we attempted to use our *S. latifolia* 454 data for repeat comparison between male and female genomes, we encountered up to three-fold differences in the abundance of some major repeat families; however, these differences were not confirmed experimentally using FISH and Southern blotting (data not shown). Thus, we switched to using Illumina sequencing for repeat quantification purposes, as it did not include the emulsion PCR step and provided significantly more frequent genome sampling (5 million reads vs. 250–420 thousand in the case of 454 sequencing). The inclusion of known amounts of two control DNA sequences demonstrated that three-fold differences in their concentrations are well distinguished and that almost all *S. latifolia* repeats differ to a much smaller extent between male and female genomes (Fig. 3). The only exception was CL398/STAR-Y, the deletion variant of the STAR-C satellite sequence, which is predominantly located on the Y chromosome (Fig. 5B).

The finding of apparent rDNA enrichment in the female DNA revealed a limitation of our quantification procedure, related to the GC content of the analyzed sequences. The lower efficiency in sequencing GC-rich templates is a known drawback of Illumina technology [22], [37] and it is likely that the proportions of such sequences are biased in samples differing in sequencing quality. Therefore, these factors should be considered when interpreting the results of comparative analyses of such samples, and the values obtained for GC-rich templates should be verified by other methods. On the other hand, we observed no general bias in our data, as most repeats were found to have the same abundance in male and female genomes (Table 1 and Fig. 3), in agreement with previous FISH investigation of partial clones of Tekay-like and Athila-like retrotransposons and DNA transposons [8]. The only exception was a group of Ogre-like repeats that were found to be more abundant in the female genome, which is consistent with the published FISH data demonstrating depletion of these sequences from the Y chromosome [8], [35]. On the contrary, the partial enrichment of some Ty1/copia sequences on the Y chromosome reported by Cermak et al. [8] was not evident from our analysis. It should be noted, however, that the successful detection of repeats that differ in their abundance on sex chromosomes using the methodologies employed in this work depends on two key factors. First, these sequences have to be sufficiently frequent in the genome to occur multiple times in our low-pass sequencing data, in order to be identified as repeats and reliably quantified (therefore we quantified only the repeats representing at least 0.005% of the genome, Fig. 3). Second, in cases in which the repeat also occurs on autosomes, the difference in its proportions between sex chromosomes should be relatively large in order to be reliably detected when analyzing total genomic DNA. For example, given that the X and Y chromosomes represent 7.5% and 10% of the *S. latifolia* genome, respectively [2], [25], then a ten-fold higher density of a repeat on the Y chromosome compared to X and autosomes results in only a 1.9-fold higher abundance of the repeat in the male total nuclear DNA compared to the female. Smaller differences are proportionally more difficult to detect in such cases; on the other hand, sex chromosome-specific or highly enriched repeats such as CL398/STAR-Y that have no or relatively few copies on autosomes can be reliably revealed. In the light of these considerations, the results presented here mainly demonstrate that evolutionarily young *S. latifolia* sex chromosomes do not contain large amounts of chromosome-specific repeats as is the case of the Y chromosomes in *Rumex acetosa* [38]. However, the process of repeat accumulation and/or differentiation is already evident in the case of some satellite repeats. The origin of the CL398/STAR-Y [8] repeats by deletion of part of the STAR-C monomer sequence probably preceded its amplification on the Y chromosome, as judged from the remnants of the STAR-C array co-localizing with the major CL398/STAR-Y signal. It is notable that neither of these signals is localized in the centromere of Y, contrary to the centromeric localization of STAR-C on chromosome X and all autosomes (Fig. 5A,B). This could be due to rearrangement events during the evolution of the Y chromosome, as also suggested by the presence of telomere-like sequences in its centromeric region [39]. The three tested subfamilies of TRAYC-like satellites (CL156 and two variants of CL68) also showed localization patterns biased towards the sex chromosomes, although some of their loci also occurred on autosomes. The observed signals of CL68 and CL156 consensus sequences only partially co-localized, suggesting that most of these repeat arrays are predominantly composed of a single subfamily only. However, the 68H2 subfamily mainly co-localized with one specific locus of 68H1 on both the X and Y chromosomes, again suggesting its origin from the repeat arrays localized on the sex chromosomes.

## Materials and Methods

### Plant material

Seeds of *Silene latifolia* ecotypes “MAV3xBystrc” (used for genomic DNA isolation) and “Bystrc” (used for chromosome preparation) were obtained from the seed collection of the Institute of Biophysics, Brno, Czech Republic. Plants were grown in the greenhouse until flower development in order to distinguish male and female individuals. Total genomic DNA was extracted from the leaves as described by Dellaporta et al. [40].

### 454 sequencing and clustering-based repeat identification

Sequencing of randomly sheared *S. latifolia* total genomic DNA was performed by 454 Life Sciences (Branford, CT, USA) using a 454 GS FLX instrument. Sequencing was run separately for samples of male and female DNA, yielding a total of 295,758 and 455,813 reads, respectively. The reads were preprocessed using custom BioPerl (http://www.bioperl.org) scripts to remove artificially duplicated reads and remnants of linkers. The reads from male and female genomes were then combined and subjected to graph-based clustering analysis as described by Novak et al. [15] to identify groups of reads representing repetitive elements. Briefly, the analysis consisted of all-to-all comparison of 454 reads using mgblast [41] and representation of pair-wise sequence similarities exceeding the specified threshold (90% similarity over 55% of the shorter sequence length) as edges in a virtual graph connecting the similar reads represented by graph nodes. The graph structure was analyzed using custom-made R (http://www.rproject.org) programs in order to detect clusters of frequently connected nodes (communities) representing groups of similar sequences. These clusters, corresponding to families of genomic repeats, were separated and analyzed with respect to their similarity to known repeats using RepeatMasker (http://www.repeatmasker/org) and BLAST [42] search against GenBank databases http://www.ncbi.nlm.nih.gov/genbank and against our database of plant mobile element protein sequences. Structural features like tandem subrepeats within the read and contig sequences were identified using DOTTER [43]. Graph layouts of individual clusters were examined interactively using the SeqGrapheR program [15].

The classification of LTR retrotransposons into established lineages and clades [18], [34], [44] based on the phylogenetic relationships of their reverse transcriptase (RT) protein domains was achieved using the RT domains extracted from assembled contig sequences. The domains were identified using the FASTY program [45], employing our in-house database of the RT domains of known plant retrotransposons. Multiple alignment of identified RT sequences was carried out using MUSCLE [46]. The alignment was used to build phylogenetic trees using the neighbor-joining method, employing observed evolutionary distances implemented in the SeaView program [47]. The trees were drawn and edited using the FigTree program (http://tree.bio.ed.ac.uk/software/figtree/).

Repeat-specific databases of 454 reads were assembled by combining the reads from clusters assigned to the same type or family of repeats. They were converted to BLAST databases [42] and employed for repeat annotation in genomic BAC sequences using the PROFREP server (http://w3lamc.umbr.cas.cz/profrep/public/). The PROFREP similarity search was conduced using blastn with the e-value cutoff set to 1e^−15^.

### Illumina sequencing and repeat quantification

Single-end Illumina sequencing of the male and female total genomic DNAs was performed by Creative Genomics (Shirley, NY, USA). A total of 18,593,581 and 26,834,102 reads were obtained from the male and female sample, respectively. In order to avoid read positions with more frequent sequencing errors and extract only high-quality read fragments (read tags), 36-nucleotide reads were trimmed to 30 nucleotides by extracting positions 3–32 and by selecting fragments with base call quality scores of 10 over at least 90% of their sequences. The sequencing quality analysis, read trimming and quality filtering were performed using the FASTX Toolkit (http://hannonlab.cshl.edu/fastx_toolkit/). Random selection of 5 million tags for further analysis was performed using custom R script. In order to use Illumina sequence tags for repeat quantification, they were assigned to 454 read clusters representing individual repeats based on their sequence similarity to the reads within the clusters. The similarities were detected using the PatMaN program [48], allowing up to two mismatches, including gaps (the gaps were allowed in order to compensate for homopolymer sequencing errors in the 454 reads). Each tag was mapped to a maximum of one cluster, based on its best similarity detected among 454 reads. Repeat proportions in the genome were then calculated from the numbers of Illumina tags mapped to individual clusters.

### Characterization of satellite repeats

Identification of the most conserved sequence variants and consensus monomer reconstruction of satellite repeats were conducted using *k*-mer frequency analysis as described previously [23]. The analysis used the Illumina sequence tags associated with the clusters containing satellite repeats and was run for each cluster separately. The lengths of the analyzed *k*-mers ranged between 10 and 17 nucleotides and the optimal conditions used for sequence reconstructions differed between repeat families (see Fig. S4 legend for detailed information). The reconstructed sequences were used to design the oligonucleotide hybridization probes listed in Table 2; their positions with respect to the reconstructed monomer sequences are shown in Fig. 4. The probes were synthesized by Generi Biotech (Hradec Králové, Czech Rep.), with the exception of the locked nucleic acid-modified oligonucleotides which were purchased from Exiqon (Vedbaek, Denmark).

### Fluorescence in situ hybridization (FISH)

In order to synchronize germinating seeds of *S. latifolia*, the DNA polymerase inhibitor aphidicolin was added for 12 h, and mitoses were then accumulated with oryzalin as described in Siroky et al. [21]. Slides were preheated at 60°C for 30 min, treated with 100 µg/ml RNase A (Sigma) in 2xSSC (0.3 M NaCl, 0.03 M sodium citrate, pH 7) for 1 h at 37°C, washed three times for 5 min in 2xSSC, treated with 5 mg/ml pepsin (Sigma) in 0.01 N HCl for 10 min at 37°C, washed as before, postfixed in 3.7% formaldehyde (Merck) in 1xPBS for 10 min, washed again and dehydrated in increasing ethanol series (70, 70, 96% ethanol, 5 min each). Slide denaturation was performed in 7∶3 (v/v) formamide:2xSSC for 2 min at 72°C. Slides were immediately dehydrated through 50, 70, and 100% ethanol (−20°C), and air-dried. The hybridization mixture contained 50% formamide (v/v, Sigma ULTRA), 10% dextran sulfate (w/v, Sigma), 2xSSC, and 0.4 µM oligonucleotide probe. The mixture was denatured by incubation at 75°C for 10 min and immediately placed on ice. Typically, 25 µl was applied per slide, covered with a plastic coverslip and hybridized for 18 h in a moist chamber. The hybridization temperature was 37°C for all probes except 12H5, which was hybridized at 28°C. Post-hybridization washing was carried out using the following steps: 2xSSC (42°C, twice for 5 min), 0.1xSSC/30% formamide (32°C, twice for 5 min), 2xSSC (RT, twice for 5 min), 4Xssc+0.1% Tween 20 (RT, 7 min). Slides were counterstained with 4',6-diamidino-2-phenylindole (DAPI, 0.5 µg/ml) in Vectashield (Vector). Images were captured using a charge-coupled device (CCD) camera and ISIS software (MetaSystems).

### Electronic resources

The original sequence data from both 454 and Illumina sequencing has been deposited to the Sequence Read Archive under the study accession number ERP000762 (http://www.ebi.ac.uk/ena/data/view/ERP000762). Pre-processed sets of reads (454 reads after duplicate removal, and trimmed and quality-filtered Illumina reads) used to perform the bioinformatic analyses are available from the author's web page (http://w3lamc.umbr.cas.cz/lamc/resources.php). This page also includes links to files containing lists of 454 reads assigned to individual clusters, contigs assembled from the 454 data, outputs for various analyses performed for read and contig sequences from individual clusters (these results are grouped in separate directories, each corresponding to one cluster) and a set of BLAST databases representing 454 reads derived from various lineages/families of *S. latifolia* repetitive elements. These libraries can also be directly searched using the PROFREP server (http://w3lamc.umbr.cas.cz/profrep/public/).

## Supporting Information

Figure S1 **Graph layouts and assignment to repeat families for the largest sequence clusters identified in the** ***S. latifolia*** **454 sequencing data.**
(PDF)

Figure S2 **Phylogenetic analysis of** ***S. latifolia*** **LTR-retrotransposons based on RT sequences detected in contigs assembled from 454 data.**
(PDF)

Figure S3 **Analysis of rDNA sequencing bias related to GC content of template DNA.**
(PDF)

Figure S4 **Reconstruction of satellite repeats based on** ***k*** **-mer frequency analysis in Illumina sequencing data.**
(PDF)
