# Correction: Next Generation Sequencing-Based Analysis of Repetitive DNA in the Model Dioceous Plant *Silene latifolia*

**DOI:** 10.1371/annotation/4ccaacb2-92d7-445a-87da-313cedf18feb

**Published:** 2011-12-13

**Authors:** Jiří Macas, Eduard Kejnovský, Pavel Neumann, Petr Novák, Andrea Koblížková, Boris Vyskot

The word dioecious was misspelled in the title, abstract, citation, and text. 

(1) The correct title is, "Next Generation Sequencing-Based Analysis of Repetitive DNA in the Model Dioecious Plant *Silene latifolia*" 

(2) The word dioecious was misspelled in the abstract. 

(3) The word Dioecious was misspelled in the citation. The correct citation is "Macas J, Kejnovský E, Neumann P, Novák P, Koblížková A, et al. (2011) Next Generation Sequencing-Based Analysis of Repetitive DNA in the Model Dioecious Plant *Silene latifolia*. PLoS ONE 6(11): e27335. doi:10.1371/journal.pone.0027335" 

(4) The word dioecious was misspelled in the text. 

