# Supplementary material for: Next Generation Sequencing-Based Analysis of Repetitive DNA in the Model Dioceous Plant Silene latifolia
Source: PLoS One. 2011 Nov 9;6(11):e27335. doi: 10.1371/journal.pone.0027335 (PMC3212565; doi:10.1371/journal.pone.0027335)
Supplement: Figure S1 — Graph layouts and assignment to repeat families for the largest sequence clusters identified in the S. latifolia 454 sequencing data. (PDF) [file pone.0027335.s001.pdf]

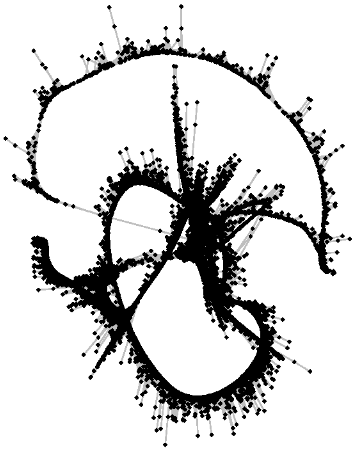

CL1

Ty1−copia/Angela
Number of reads:42110 (6.3%)
Number of pairs: 5574882
Density: 0.006288
Mean edge weigth: 88269
Max. degree: 1443

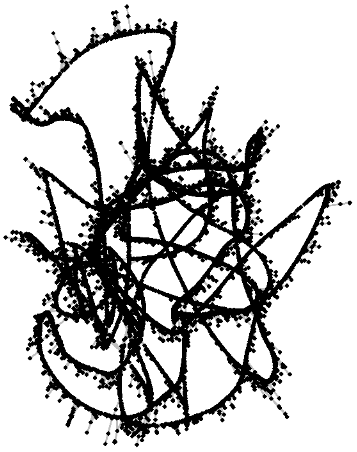

CL2

plastid DNA
Number of reads:34044 (5.1%)
Number of pairs: 957451
Density: 0.001652
Mean edge weigth: 157260
Max. degree: 140

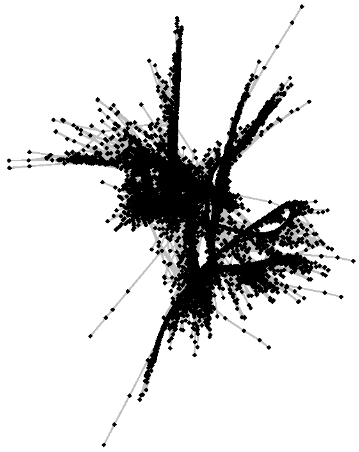

CL3

Ty3−gypsy/Athila
Number of reads:31762 (4.7%)
Number of pairs: 3463680
Density: 0.006867
Mean edge weigth: 86300
Max. degree: 1150

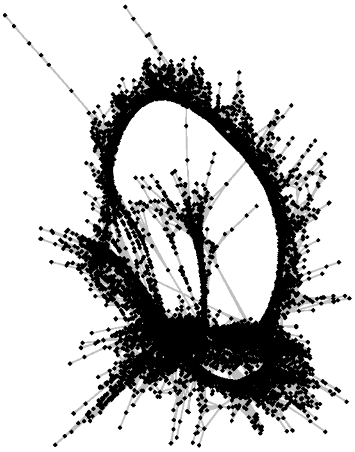

CL4

Ty3−gypsy/Tekay
Number of reads:27480 (4.1%)
Number of pairs: 1309810
Density: 0.003469
Mean edge weigth: 78301
Max. degree: 861

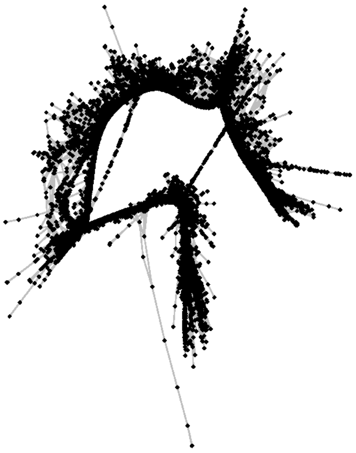

CL5

Ty3−gypsy/Ogre
Number of reads:24676 (3.7%)
Number of pairs: 2122772
Density: 0.006973
Mean edge weigth: 82319
Max. degree: 1312

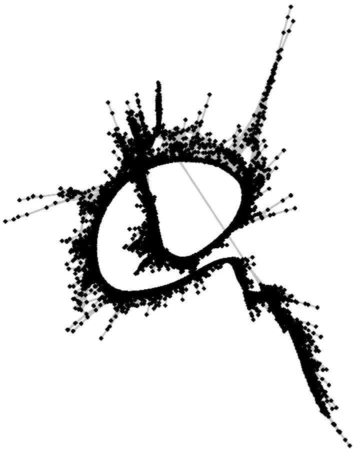

CL6

Ty3−gypsy/Ogre
Number of reads:21369 (3.2%)
Number of pairs: 1205001
Density: 0.005278
Mean edge weigth: 86789
Max. degree: 590

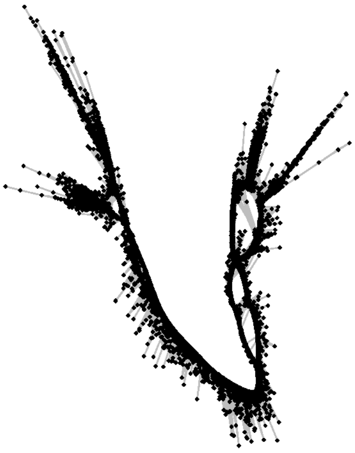

CL7

Ty1−copia/Angela
Number of reads:20948 (3.1%)
Number of pairs: 2495607
Density: 0.01137
Mean edge weigth: 92990
Max. degree: 603

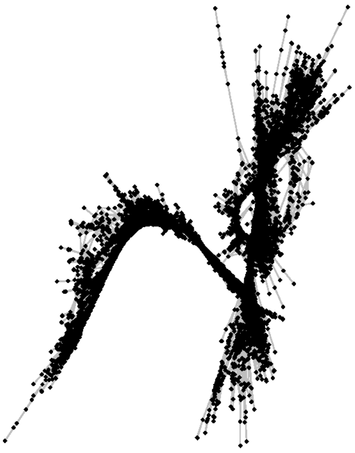

CL8

Ty3−gypsy/Ogre
Number of reads:16106 (2.4%)
Number of pairs: 1041280
Density: 0.008029
Mean edge weigth: 84269
Max. degree: 831

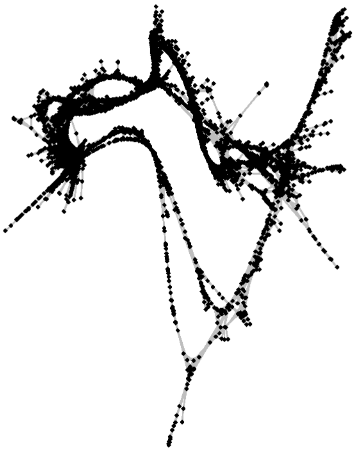

CL9

Ty3−gypsy/Tat/Retand
Number of reads:10138 (1.5%)
Number of pairs: 238952
Density: 0.00465
Mean edge weigth: 78567
Max. degree: 305

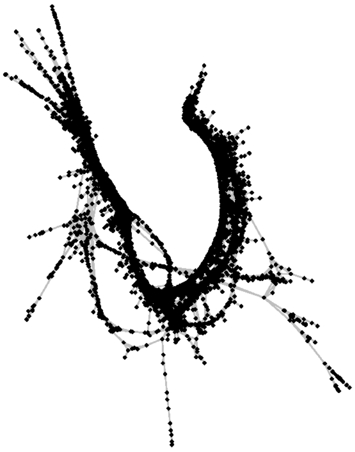

CL10

Ty3−gypsy/Athila
Number of reads:9643 (1.4%)
Number of pairs: 190083
Density: 0.004089
Mean edge weigth: 77025
Max. degree: 332

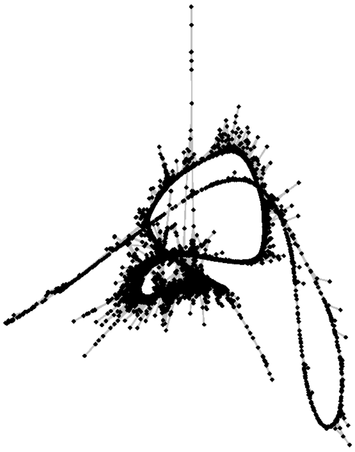

CL11

Ty3−gypsy/Ogre
Number of reads:9574 (1.4%)
Number of pairs: 456941
Density: 0.009971
Mean edge weigth: 103220
Max. degree: 389

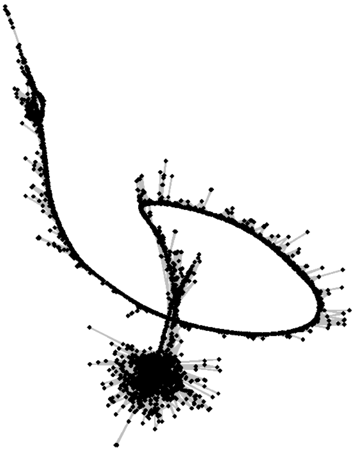

CL12

rDNA+satellite(X43.1)
Number of reads:9285 (1.4%)
Number of pairs: 2270308
Density: 0.05267
Mean edge weigth: 80392
Max. degree: 2308

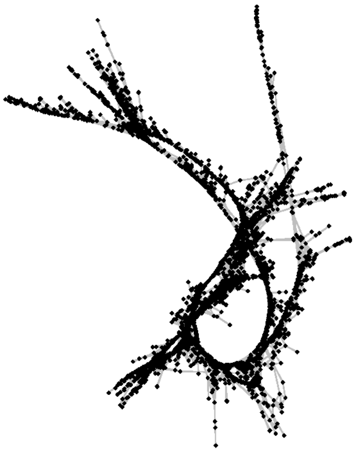

CL13

Ty3–gypsy
Number of reads:6023 (0.9%)
Number of pairs: 199713
Density: 0.01101
Mean edge weigth: 102080
Max. degree: 324

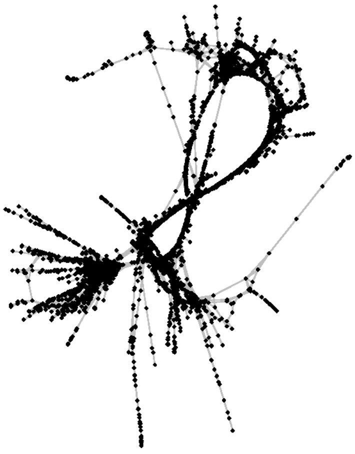

CL14

Ty3–gypsy
Number of reads:5947 (0.88%)
Number of pairs: 92260
Density: 0.005218
Mean edge weigth: 88549
Max. degree: 153

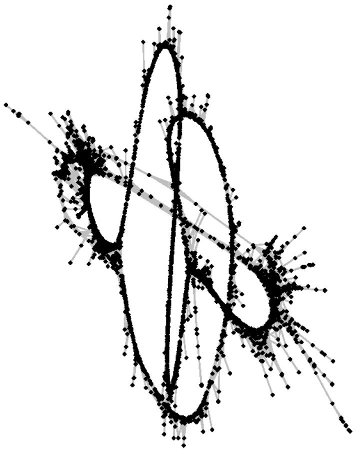

CL15

Ty1–copia/Angela
Number of reads:5170 (0.77%)
Number of pairs: 154945
Density: 0.0116
Mean edge weigth: 98653
Max. degree: 224

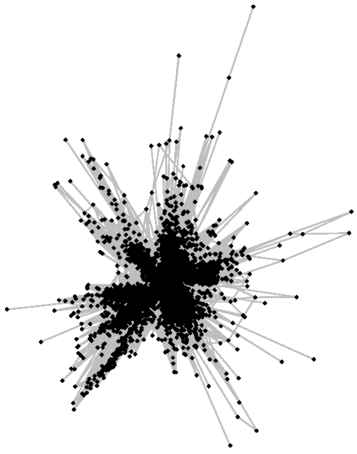

CL16

satellite (STAR)
Number of reads:5162 (0.77%)
Number of pairs: 542665
Density: 0.04074
Mean edge weigth: 53335
Max. degree: 2073

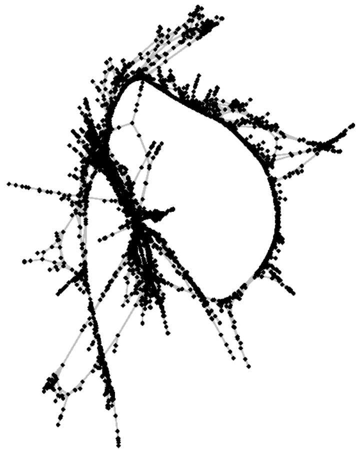

CL17

Ty3–gypsy/Tekay
Number of reads:5161 (0.77%)
Number of pairs: 61917
Density: 0.00465
Mean edge weigth: 80566
Max. degree: 243

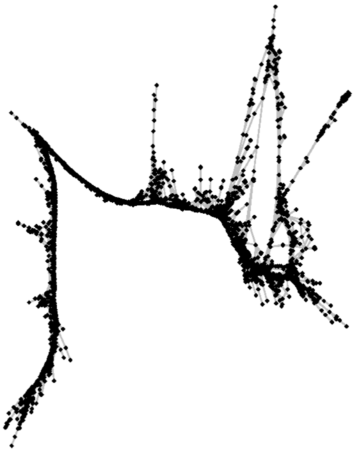

CL18

Ty1–copia/Maximus–SIRE
Number of reads:3941 (0.59%)
Number of pairs: 72674
Density: 0.009361
Mean edge weigth: 90927
Max. degree: 189

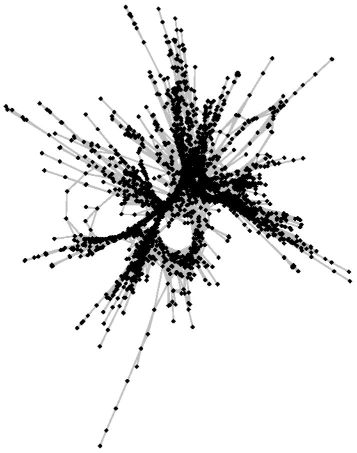

CL19

unclassified
Number of reads:3927 (0.58%)
Number of pairs: 111582
Density: 0.01447
Mean edge weigth: 80202
Max. degree: 278

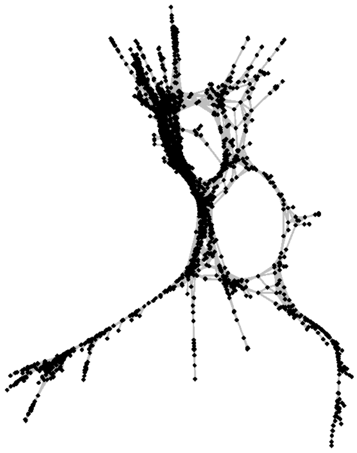

CL20

Ty3–gypsy/Athila
Number of reads:3676 (0.55%)
Number of pairs: 69538
Density: 0.01029
Mean edge weigth: 89717
Max. degree: 155

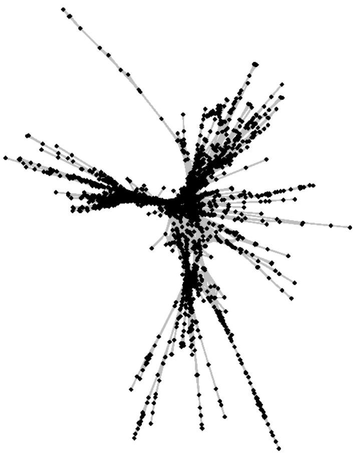

CL21

unclassified
Number of reads:3228 (0.48%)
Number of pairs: 96636
Density: 0.01855
Mean edge weigth: 69231
Max. degree: 324

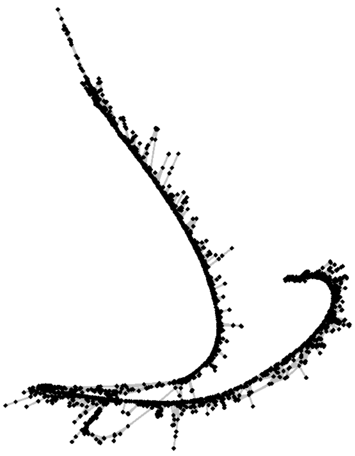

CL22

DNA transp/CACTA
Number of reads:3076 (0.46%)
Number of pairs: 64548
Density: 0.01365
Mean edge weigth: 111600
Max. degree: 91

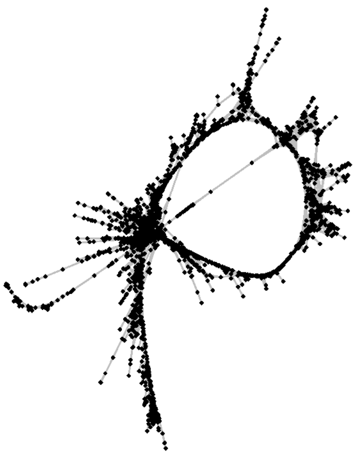

CL23

Ty3–gypsy/Athila
Number of reads:2982 (0.44%)
Number of pairs: 42596
Density: 0.009584
Mean edge weigth: 84870
Max. degree: 249

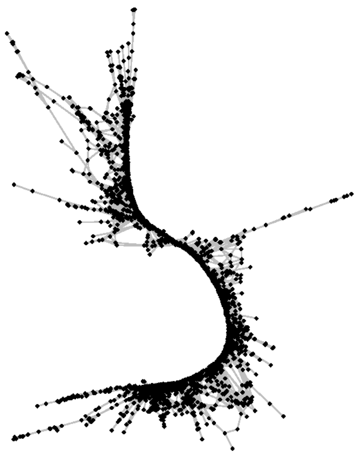

CL24

Ty3–gypsy/Athila
Number of reads:2926 (0.44%)
Number of pairs: 75881
Density: 0.01773
Mean edge weigth: 81911
Max. degree: 140

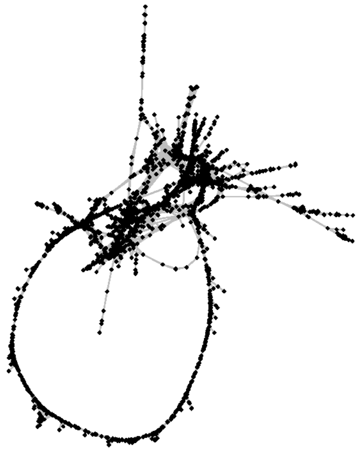

CL25

Ty3−gypsy/Tekay
Number of reads:2926 (0.44%)
Number of pairs: 32997
Density: 0.007711
Mean edge weigth: 96537
Max. degree: 85

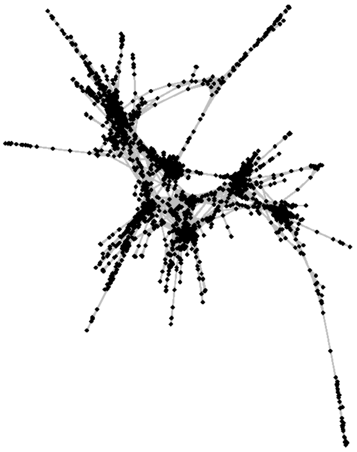

CL26

Ty1−copia/Angela
Number of reads:2842 (0.42%)
Number of pairs: 25196
Density: 0.006241
Mean edge weigth: 83718
Max. degree: 121

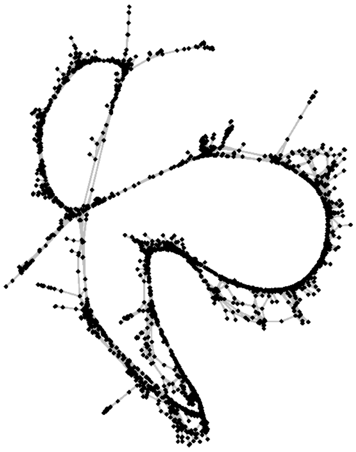

CL27

Ty3−gypsy/Tat
Number of reads:2561 (0.38%)
Number of pairs: 26561
Density: 0.008103
Mean edge weigth: 76975
Max. degree: 71

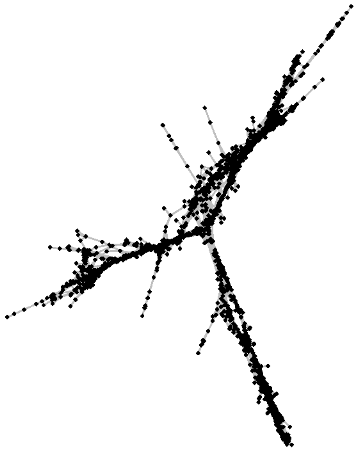

CL28

Ty1−copia/Angela
Number of reads:2254 (0.34%)
Number of pairs: 31897
Density: 0.01256
Mean edge weigth: 73806
Max. degree: 140

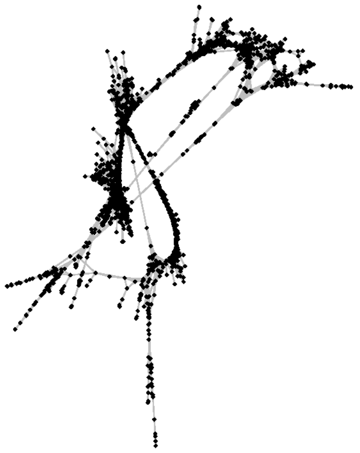

CL29

unclassified
Number of reads:2165 (0.32%)
Number of pairs: 32545
Density: 0.01389
Mean edge weigth: 78034
Max. degree: 203

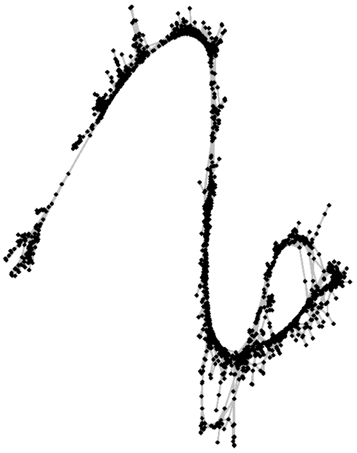

CL30

Ty3−gypsy/Ogre
Number of reads:2109 (0.31%)
Number of pairs: 23725
Density: 0.01067
Mean edge weigth: 72589
Max. degree: 93

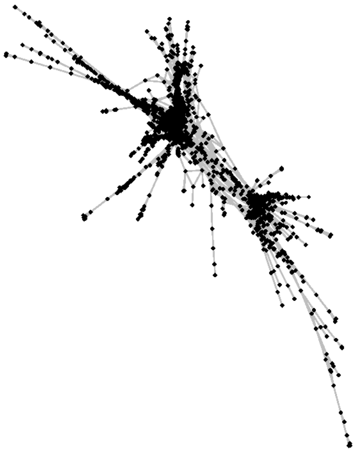

CL31

unclassified
Number of reads:2103 (0.31%)
Number of pairs: 44915
Density: 0.02032
Mean edge weigth: 100260
Max. degree: 258

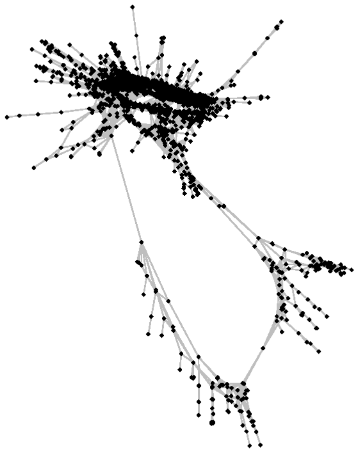

CL32

Ty3−gypsy/Ogre
Number of reads:2092 (0.31%)
Number of pairs: 37283
Density: 0.01705
Mean edge weigth: 77734
Max. degree: 150

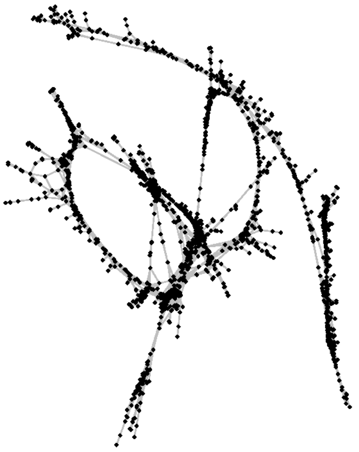

CL33

Ty3−gypsy/Athila
Number of reads:2014 (0.3%)
Number of pairs: 16060
Density: 0.007923
Mean edge weigth: 81752
Max. degree: 97

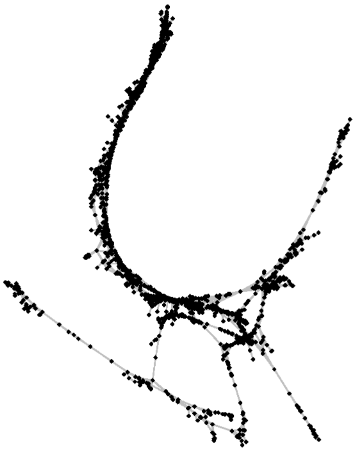

CL34

Ty1−copia/Angela
Number of reads:2007 (0.3%)
Number of pairs: 16250
Density: 0.008072
Mean edge weigth: 78077
Max. degree: 74

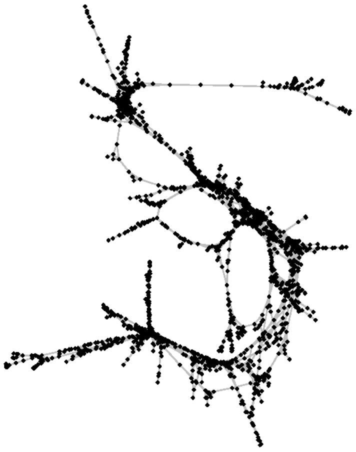

CL35

unclassified
Number of reads:1997 (0.3%)
Number of pairs: 13093
Density: 0.006569
Mean edge weigth: 81677
Max. degree: 83

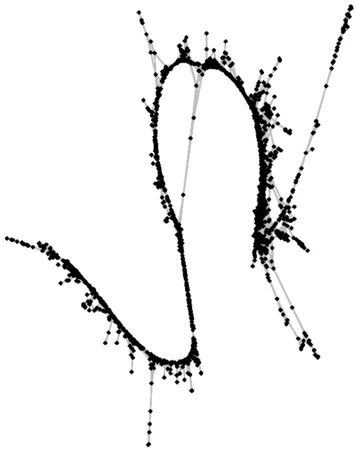

CL36

Ty1−copia/Maximus−SIRE
Number of reads:1953 (0.29%)
Number of pairs: 22869
Density: 0.012
Mean edge weigth: 102240
Max. degree: 59

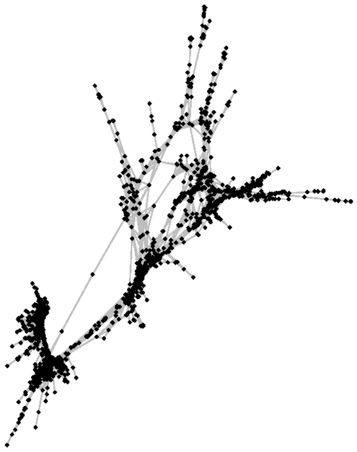

CL37

unclassified
Number of reads:1863 (0.28%)
Number of pairs: 38286
Density: 0.02207
Mean edge weigth: 76446
Max. degree: 233

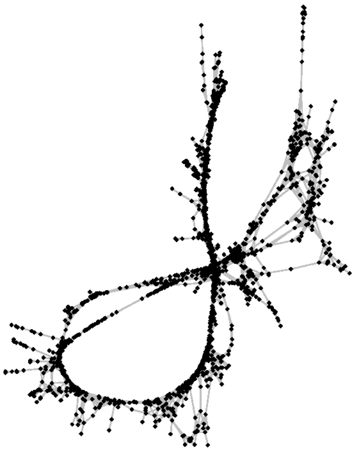

CL38

Ty3–gypsy/Tat
Number of reads:1801 (0.27%)
Number of pairs: 16573
Density: 0.01022
Mean edge weigth: 69749
Max. degree: 67

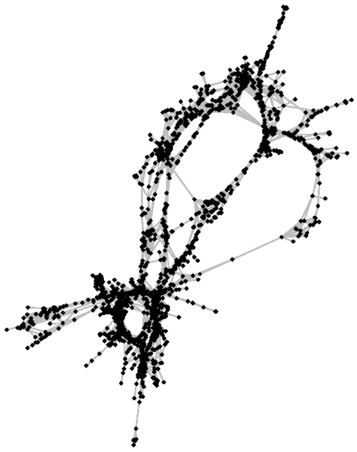

CL39

Ty3–gypsy/Tat/Retand
Number of reads:1794 (0.27%)
Number of pairs: 14273
Density: 0.008874
Mean edge weigth: 78892
Max. degree: 74

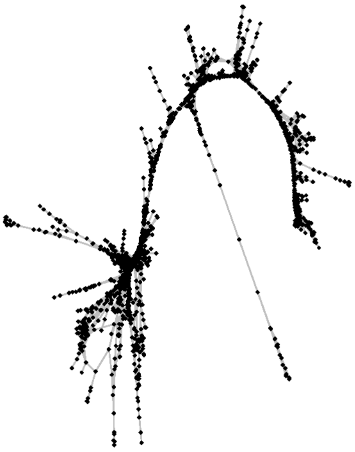

CL40

Ty3–gypsy/Ogre
Number of reads:1788 (0.27%)
Number of pairs: 21822
Density: 0.01366
Mean edge weigth: 83203
Max. degree: 155

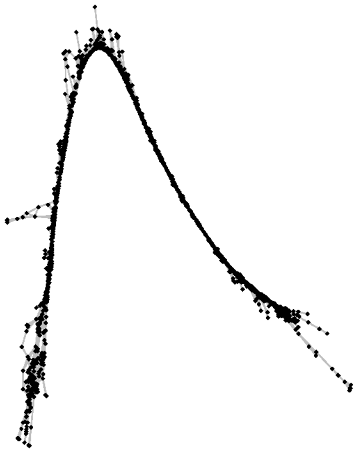

CL41

Ty3–gypsy/Tat
Number of reads:1738 (0.26%)
Number of pairs: 32122
Density: 0.02128
Mean edge weigth: 83609
Max. degree: 68

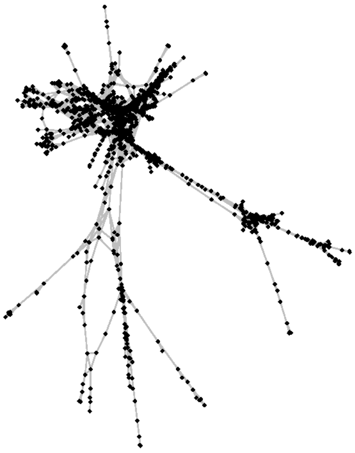

CL42

Ty3–gypsy/Athila
Number of reads:1558 (0.23%)
Number of pairs: 17993
Density: 0.01483
Mean edge weigth: 82764
Max. degree: 101

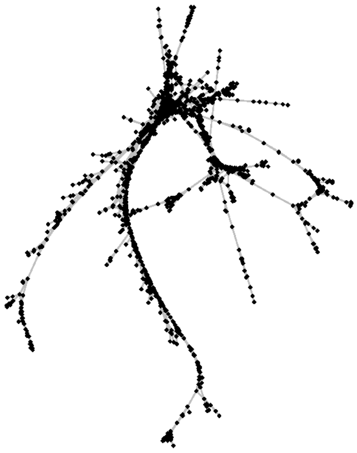

CL43

Ty3–gypsy/Athila
Number of reads:1502 (0.22%)
Number of pairs: 10415
Density: 0.009239
Mean edge weigth: 82746
Max. degree: 84

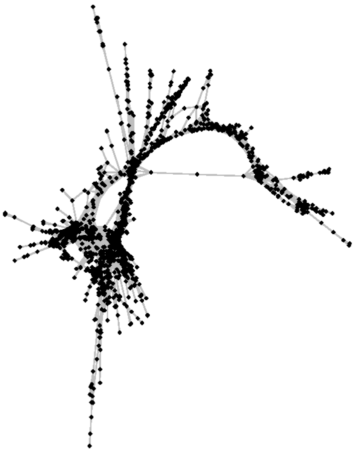

CL44

Ty3–gypsy/Athila
Number of reads:1501 (0.22%)
Number of pairs: 17485
Density: 0.01553
Mean edge weigth: 86845
Max. degree: 152

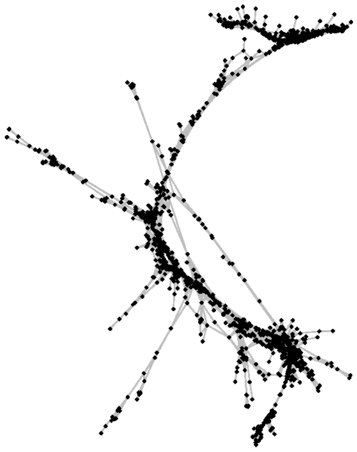

CL45

Ty3–gypsy/Tekay
Number of reads:1491 (0.22%)
Number of pairs: 11720
Density: 0.01055
Mean edge weigth: 79598
Max. degree: 64

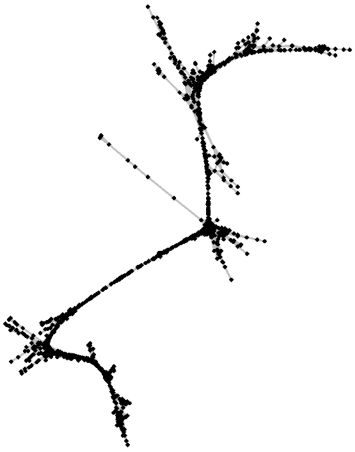

CL46

Ty1–copia/Maximus–SIRE
Number of reads:1445 (0.21%)
Number of pairs: 14550
Density: 0.01395
Mean edge weigth: 89049
Max. degree: 91

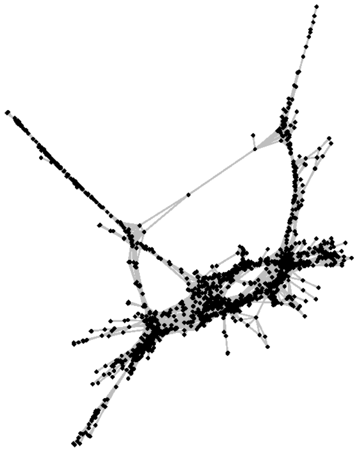

CL47

Ty3–gypsy/Athila
Number of reads:1443 (0.21%)
Number of pairs: 13457
Density: 0.01293
Mean edge weigth: 75170
Max. degree: 80

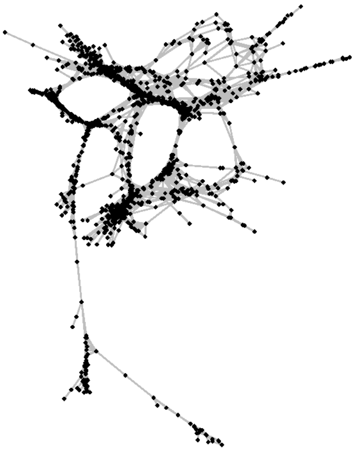

CL48

DNA transp/CACTA
Number of reads:1414 (0.21%)
Number of pairs: 25279
Density: 0.0253
Mean edge weigth: 103210
Max. degree: 133

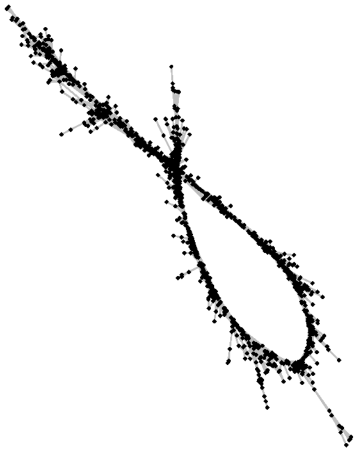

CL49

Ty3−gypsy/Ogre
Number of reads:1340 (0.2%)
Number of pairs: 13999
Density: 0.0156
Mean edge weigth: 73701
Max. degree: 83

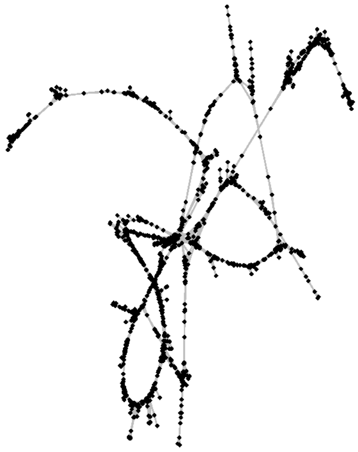

CL50

DNA transp/CACTA
Number of reads:1302 (0.19%)
Number of pairs: 7798
Density: 0.009207
Mean edge weigth: 119210
Max. degree: 43

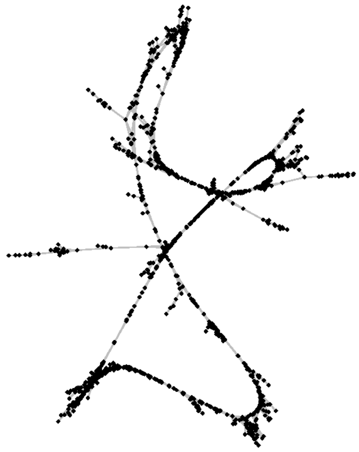

CL51

Ty3−gypsy/Tat
Number of reads:1289 (0.19%)
Number of pairs: 8383
Density: 0.0101
Mean edge weigth: 91571
Max. degree: 48

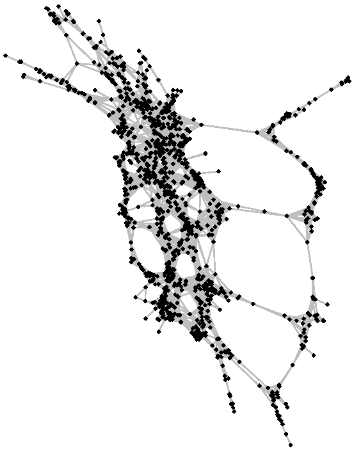

CL52

Ty3−gypsy/Athila
Number of reads:1201 (0.18%)
Number of pairs: 11516
Density: 0.01598
Mean edge weigth: 92523
Max. degree: 53

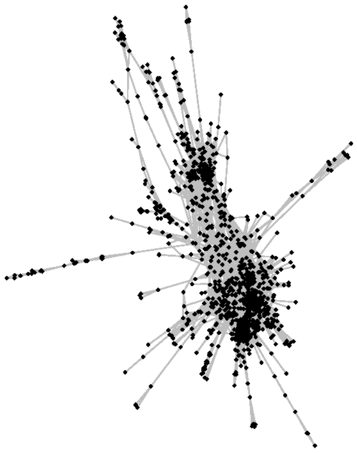

CL53

Ty1−copia/Angela
Number of reads:1200 (0.18%)
Number of pairs: 13353
Density: 0.01856
Mean edge weigth: 77737
Max. degree: 114

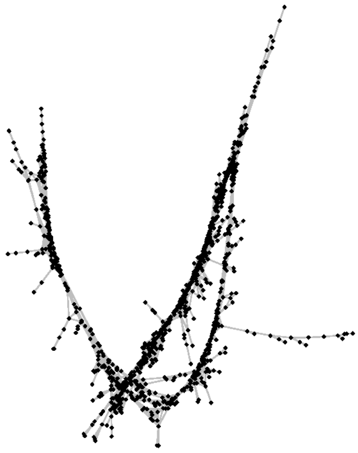

CL54

Ty3−gypsy/Tat
Number of reads:1193 (0.18%)
Number of pairs: 12308
Density: 0.01731
Mean edge weigth: 72553
Max. degree: 68

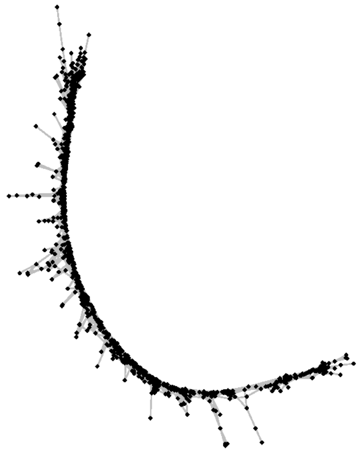

CL55

LINE
Number of reads:1092 (0.16%)
Number of pairs: 19847
Density: 0.03332
Mean edge weigth: 84055
Max. degree: 88

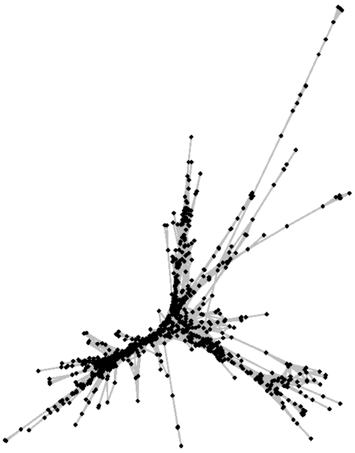

CL56

unclassified
Number of reads:1018 (0.15%)
Number of pairs: 18322
Density: 0.03539
Mean edge weigth: 80903
Max. degree: 139

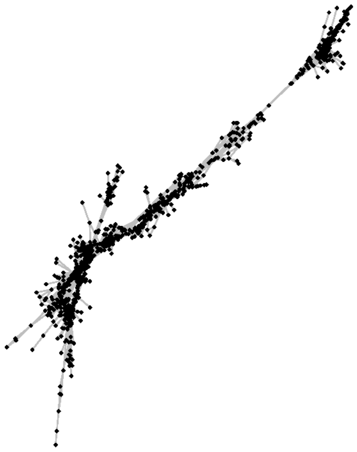

CL57

Ty3−gypsy/Ogre
Number of reads:961 (0.14%)
Number of pairs: 9060
Density: 0.01964
Mean edge weigth: 75288
Max. degree: 100

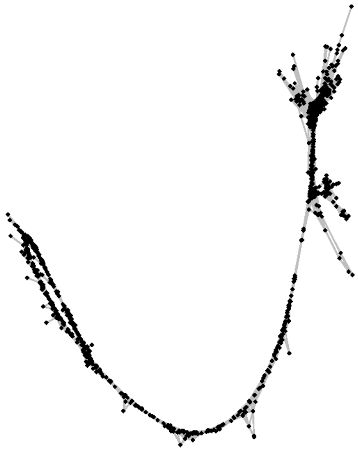

CL58

Ty3−gypsy/Tekay
Number of reads:944 (0.14%)
Number of pairs: 13822
Density: 0.03105
Mean edge weigth: 114340
Max. degree: 149

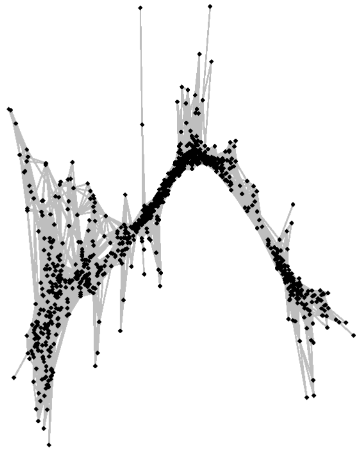

CL59

rDNA (IGS)
Number of reads:943 (0.14%)
Number of pairs: 24958
Density: 0.05619
Mean edge weigth: 122220
Max. degree: 113

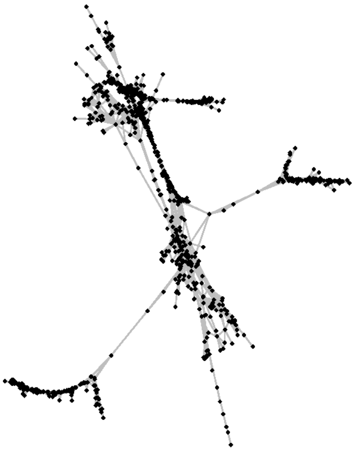

CL60

Ty3−gypsy/chrom
Number of reads:939 (0.14%)
Number of pairs: 7438
Density: 0.01689
Mean edge weigth: 94319
Max. degree: 51

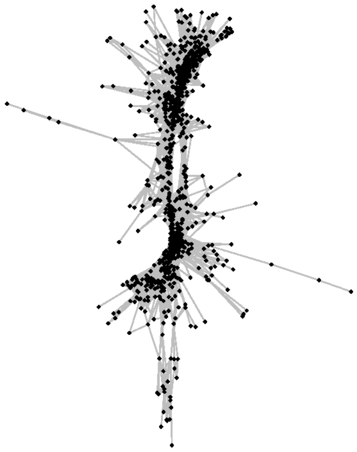

CL61

Ty3−gypsy/Ogre
Number of reads:930 (0.14%)
Number of pairs: 19670
Density: 0.04553
Mean edge weigth: 84966
Max. degree: 137

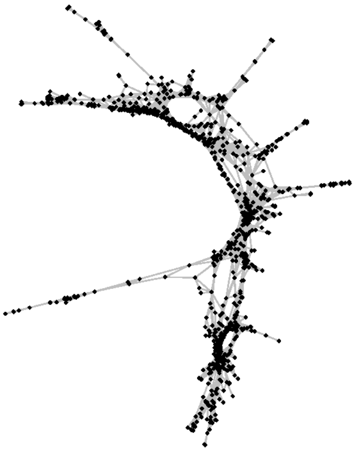

CL62

Ty3−gypsy/Tekay
Number of reads:928 (0.14%)
Number of pairs: 7613
Density: 0.0177
Mean edge weigth: 86958
Max. degree: 62

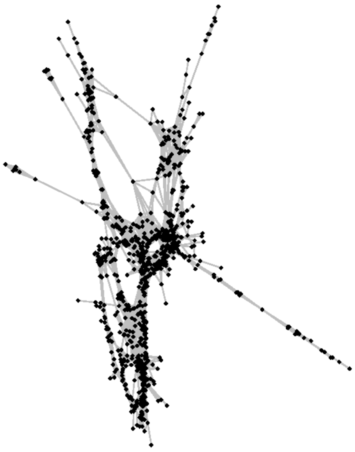

CL63

Ty3−gypsy/Athila
Number of reads:916 (0.14%)
Number of pairs: 7640
Density: 0.01823
Mean edge weigth: 91083
Max. degree: 63

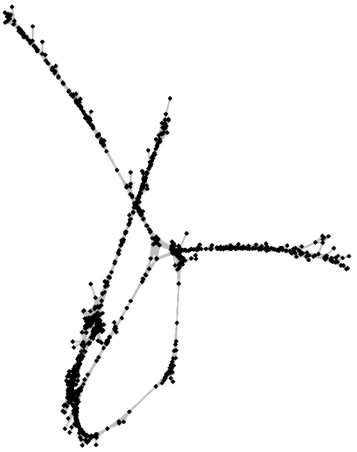

CL64

DNA transp/CACTA
Number of reads:901 (0.13%)
Number of pairs: 6714
Density: 0.01656
Mean edge weigth: 86868
Max. degree: 47

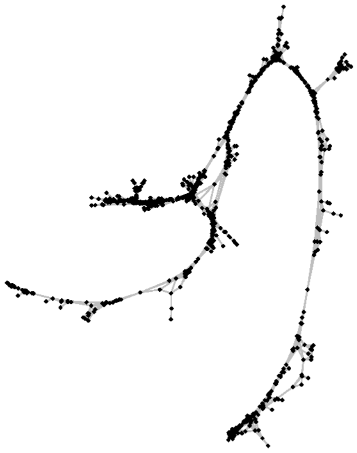

CL65

Ty1−copia
Number of reads:890 (0.13%)
Number of pairs: 7881
Density: 0.01992
Mean edge weigth: 105880
Max. degree: 58

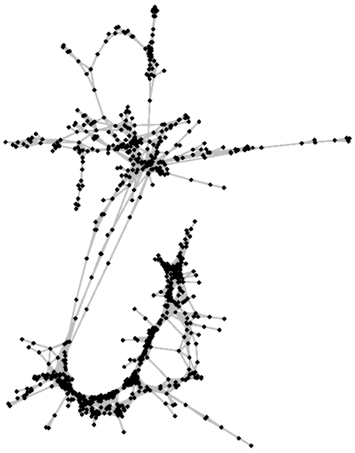

CL66

Ty3−gypsy/Tat
Number of reads:872 (0.13%)
Number of pairs: 5450
Density: 0.01435
Mean edge weigth: 84891
Max. degree: 52

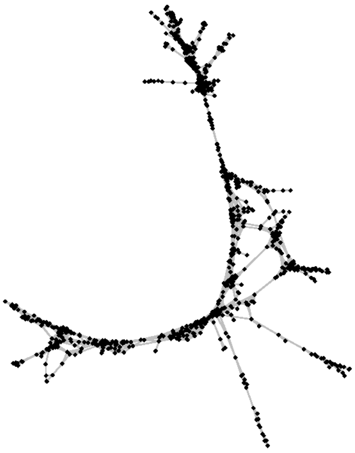

CL67

Ty3−gypsy/Athila
Number of reads:868 (0.13%)
Number of pairs: 5379
Density: 0.0143
Mean edge weigth: 86692
Max. degree: 49

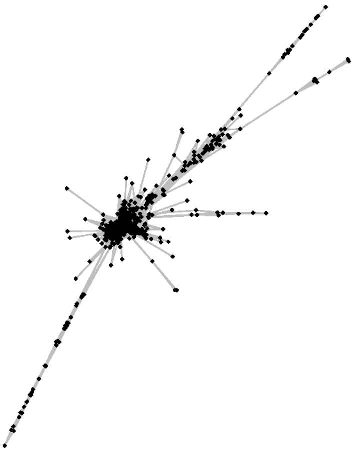

CL68

satellite/TRAYC−like
Number of reads:866 (0.13%)
Number of pairs: 70378
Density: 0.1879
Mean edge weigth: 66130
Max. degree: 463

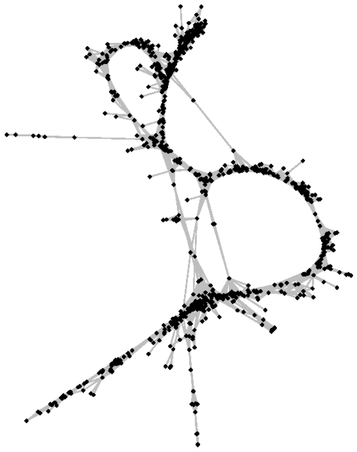

CL69

unclassified
Number of reads:848 (0.13%)
Number of pairs: 9814
Density: 0.02733
Mean edge weigth: 100250
Max. degree: 66

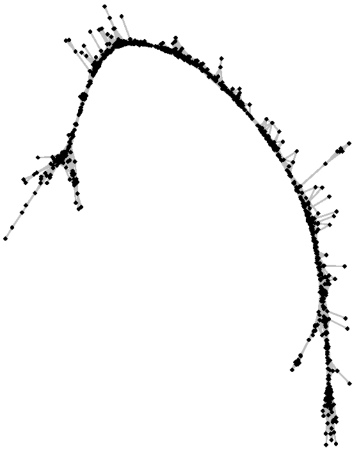

CL70

Ty3−gypsy/Athila
Number of reads:843 (0.13%)
Number of pairs: 12159
Density: 0.03426
Mean edge weigth: 93528
Max. degree: 58

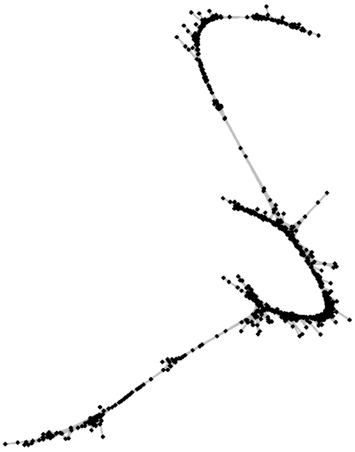

CL71

DNA transp/CACTA
Number of reads:841 (0.13%)
Number of pairs: 9755
Density: 0.02762
Mean edge weigth: 85648
Max. degree: 68

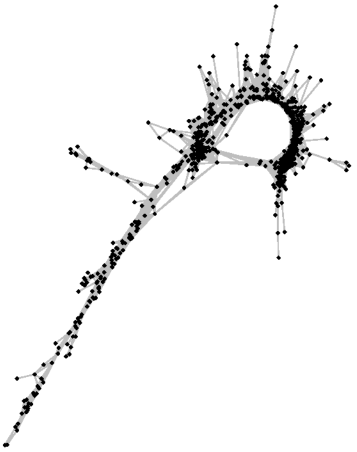

CL72

unclassified
Number of reads:728 (0.11%)
Number of pairs: 14620
Density: 0.05525
Mean edge weigth: 87173
Max. degree: 137

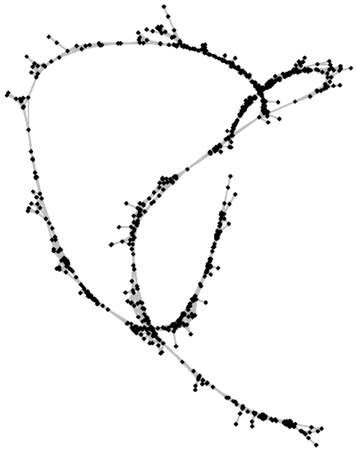

CL73

unclassified
Number of reads:720 (0.11%)
Number of pairs: 4481
Density: 0.01731
Mean edge weigth: 99737
Max. degree: 33

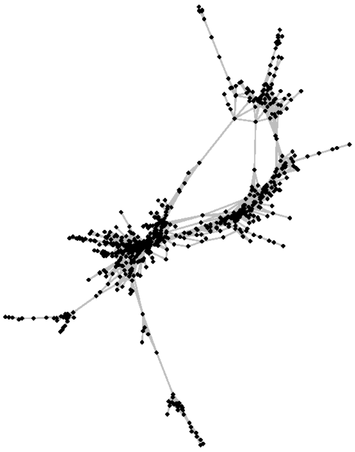

CL74

unclassified
Number of reads:701 (0.1%)
Number of pairs: 3716
Density: 0.01515
Mean edge weigth: 64952
Max. degree: 92

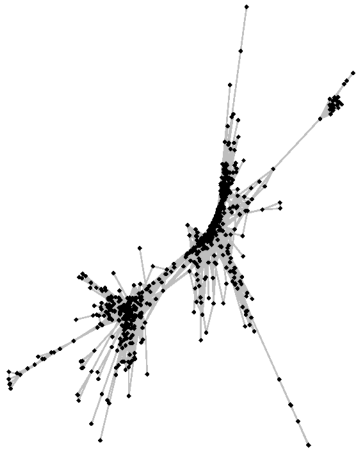

CL75

Ty3−gypsy/Athila
Number of reads:670 (0.1%)
Number of pairs: 12896
Density: 0.05754
Mean edge weigth: 78330
Max. degree: 129

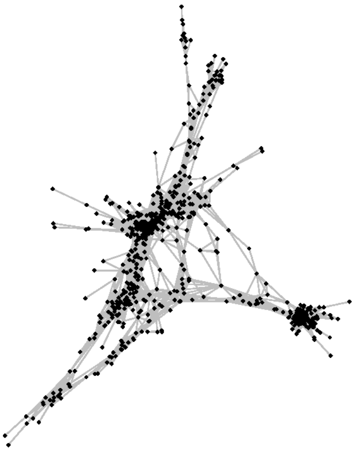

CL76

Ty3−gypsy/Ogre
Number of reads:651 (0.097%)
Number of pairs: 7226
Density: 0.03415
Mean edge weigth: 57031
Max. degree: 106

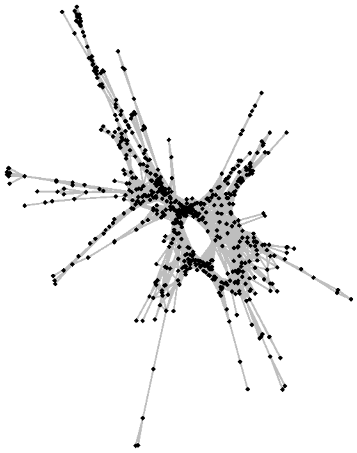

CL77

Ty3−gypsy/Athila
Number of reads:629 (0.094%)
Number of pairs: 6223
Density: 0.03151
Mean edge weigth: 93853
Max. degree: 83

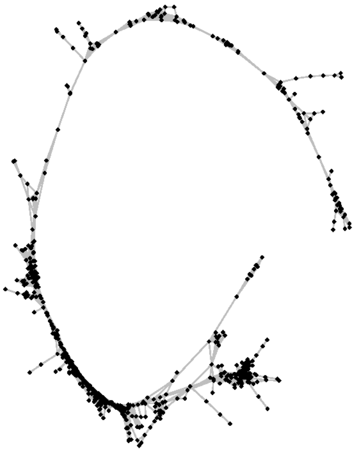

CL78

Ty3−gypsy/Athila
Number of reads:625 (0.093%)
Number of pairs: 6031
Density: 0.03093
Mean edge weigth: 82020
Max. degree: 58

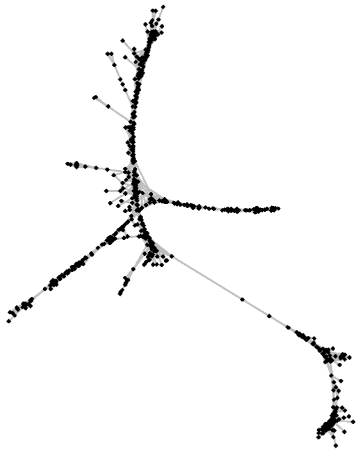

CL79

Ty1−copia/Maximus−SIRE
Number of reads:603 (0.09%)
Number of pairs: 4599
Density: 0.02534
Mean edge weigth: 87588
Max. degree: 35

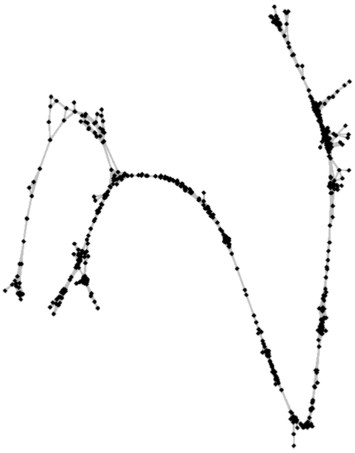

CL80

Ty3−gypsy/chrom/CRM
Number of reads:583 (0.087%)
Number of pairs: 3810
Density: 0.02246
Mean edge weigth: 102170
Max. degree: 32

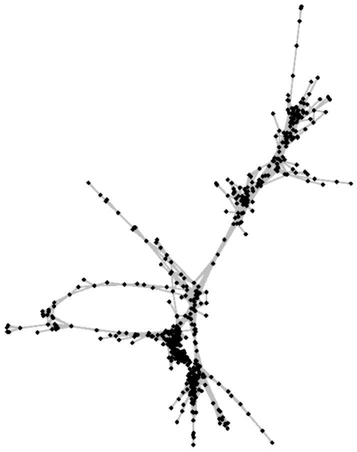

CL81

Ty1−copia/Angela
Number of reads:581 (0.086%)
Number of pairs: 4016
Density: 0.02384
Mean edge weigth: 89507
Max. degree: 45

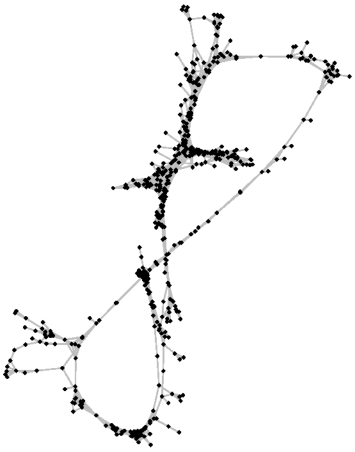

CL82

Ty3−gypsy/Athila
Number of reads:554 (0.082%)
Number of pairs: 3413
Density: 0.02228
Mean edge weigth: 81109
Max. degree: 60

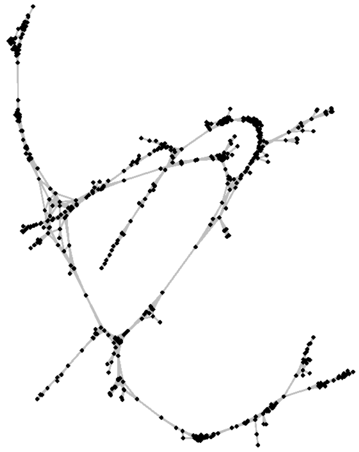

CL83

Ty3−gypsy/chrom/CRM
Number of reads:534 (0.079%)
Number of pairs: 2455
Density: 0.01725
Mean edge weigth: 120610
Max. degree: 25

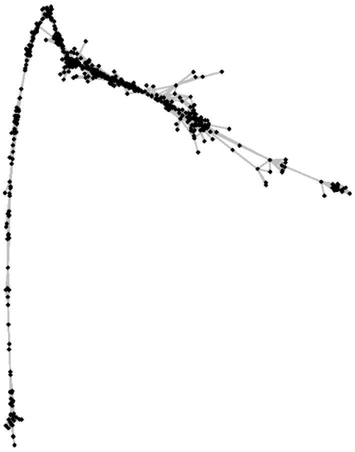

CL84

LINE
Number of reads:510 (0.076%)
Number of pairs: 4435
Density: 0.03417
Mean edge weigth: 100130
Max. degree: 48

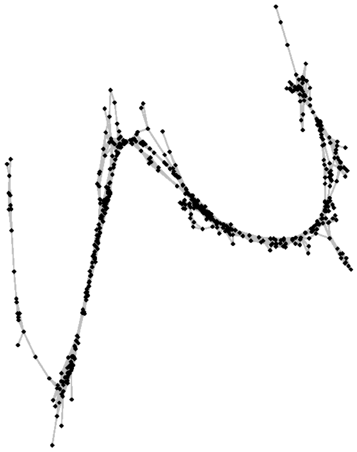

CL85

Ty1−copia/TAR
Number of reads:508 (0.076%)
Number of pairs: 3494
Density: 0.02713
Mean edge weigth: 94178
Max. degree: 40

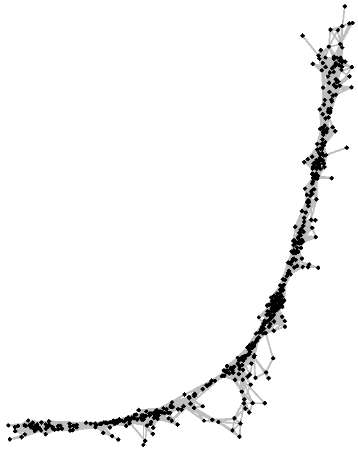

CL86

unclassified
Number of reads:499 (0.074%)
Number of pairs: 5931
Density: 0.04773
Mean edge weigth: 78689
Max. degree: 62

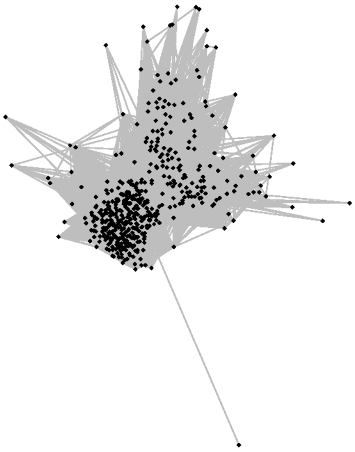

CL87

satellite (15Ssp)
Number of reads:493 (0.073%)
Number of pairs: 52280
Density: 0.4311
Mean edge weigth: 72428
Max. degree: 382

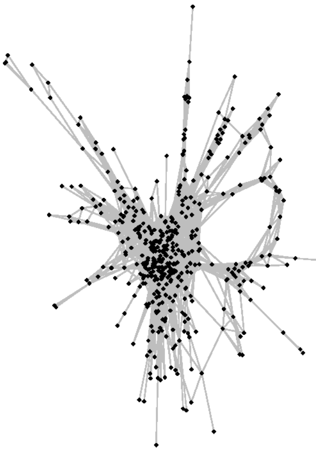

CL88

unclassified
Number of reads:451 (0.067%)
Number of pairs: 5145
Density: 0.0507
Mean edge weigth: 90470
Max. degree: 101

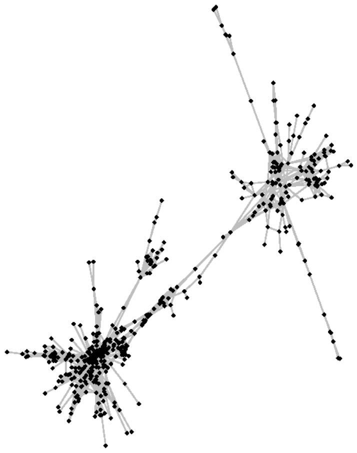

CL89

Ty3−gypsy/Ogre
Number of reads:450 (0.067%)
Number of pairs: 1858
Density: 0.01839
Mean edge weigth: 73267
Max. degree: 48

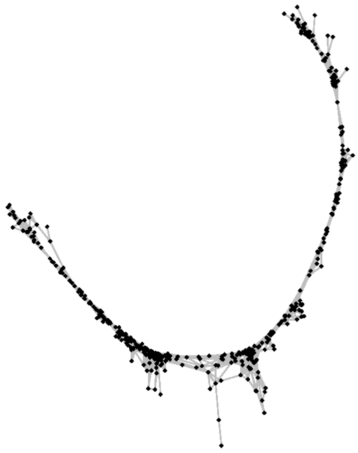

CL90

Ty1−copia/Maximus−SIRE
Number of reads:417 (0.062%)
Number of pairs: 3998
Density: 0.04609
Mean edge weigth: 107010
Max. degree: 53

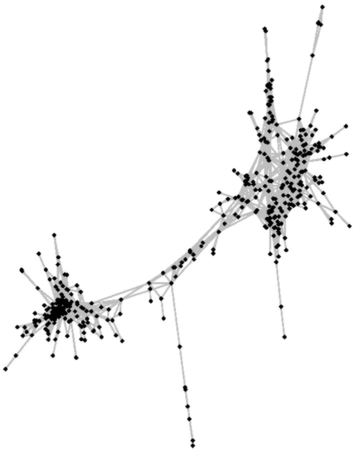

CL91

Ty1−copia/Angela
Number of reads:409 (0.061%)
Number of pairs: 2256
Density: 0.02704
Mean edge weigth: 81873
Max. degree: 60

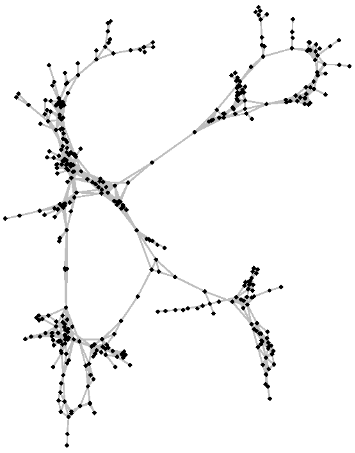

CL92

unclassified
Number of reads:397 (0.059%)
Number of pairs: 1116
Density: 0.0142
Mean edge weigth: 89377
Max. degree: 25

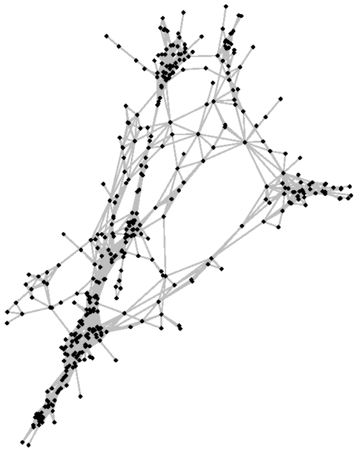

CL93

Ty3−gypsy/Ogre
Number of reads:397 (0.059%)
Number of pairs: 1609
Density: 0.02047
Mean edge weigth: 76238
Max. degree: 32

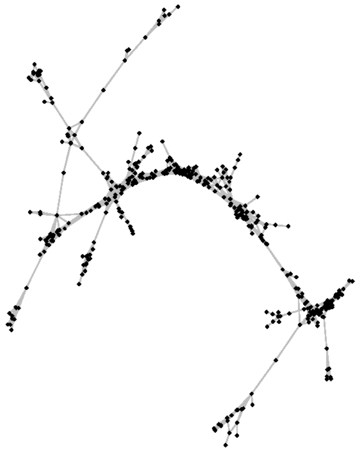

CL94

unclassified
Number of reads:391 (0.058%)
Number of pairs: 1773
Density: 0.02325
Mean edge weigth: 91780
Max. degree: 31

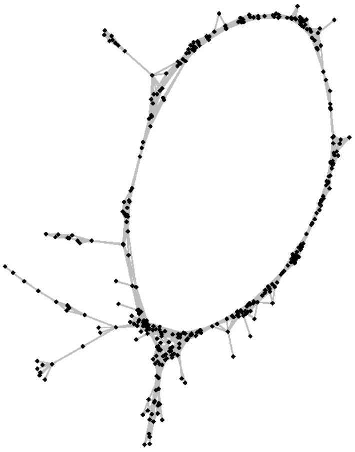

CL95

Ty3−gypsy/chrom
Number of reads:367 (0.055%)
Number of pairs: 2456
Density: 0.03657
Mean edge weigth: 103390
Max. degree: 29

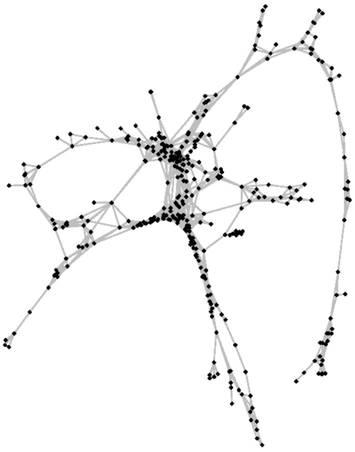

CL96

Ty3−gypsy/Athila
Number of reads:366 (0.054%)
Number of pairs: 1877
Density: 0.0281
Mean edge weigth: 110690
Max. degree: 40

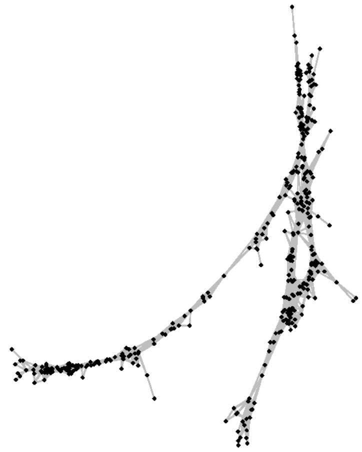

CL97

unclassified
Number of reads:361 (0.054%)
Number of pairs: 2384
Density: 0.03669
Mean edge weigth: 97663
Max. degree: 35

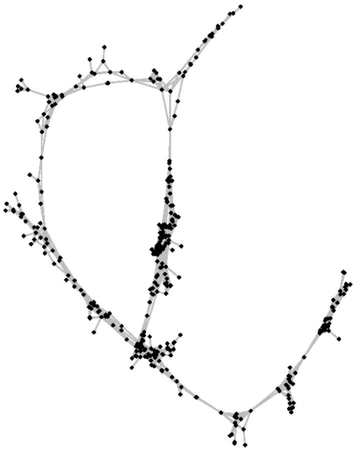

CL98

Ty3−gypsy/Athila
Number of reads:359 (0.053%)
Number of pairs: 1439
Density: 0.02239
Mean edge weigth: 72480
Max. degree: 24

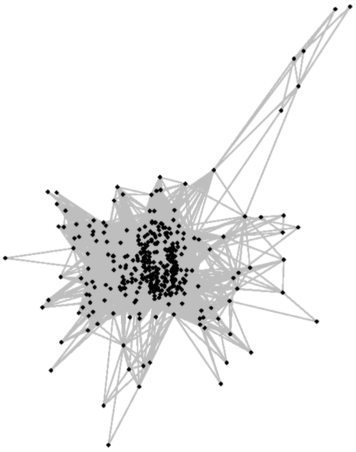

CL99

rDNA/5S
Number of reads:352 (0.052%)
Number of pairs: 17894
Density: 0.2897
Mean edge weigth: 105420
Max. degree: 181

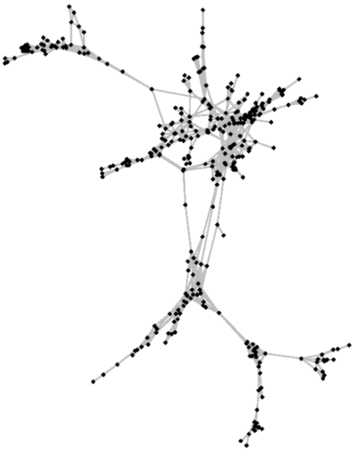

CL100

unclassified
Number of reads:351 (0.052%)
Number of pairs: 1096
Density: 0.01784
Mean edge weigth: 106720
Max. degree: 23

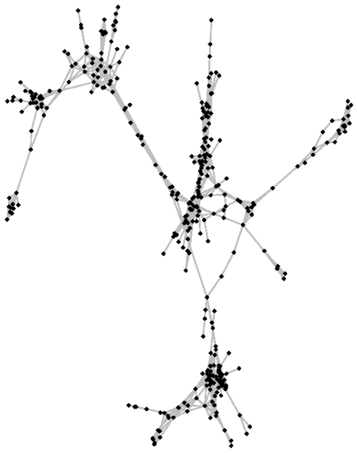

CL101

Ty3−gypsy/chrom
Number of reads:347 (0.052%)
Number of pairs: 1019
Density: 0.01697
Mean edge weigth: 67494
Max. degree: 23

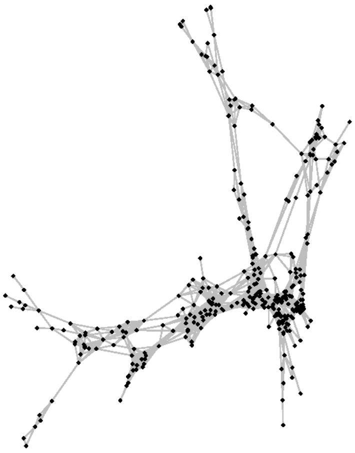

CL102

unclassified
Number of reads:334 (0.05%)
Number of pairs: 1704
Density: 0.03064
Mean edge weigth: 95403
Max. degree: 30

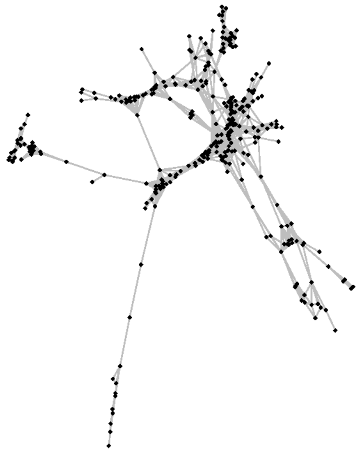

CL103

unclassified
Number of reads:320 (0.048%)
Number of pairs: 1697
Density: 0.03325
Mean edge weigth: 83379
Max. degree: 36

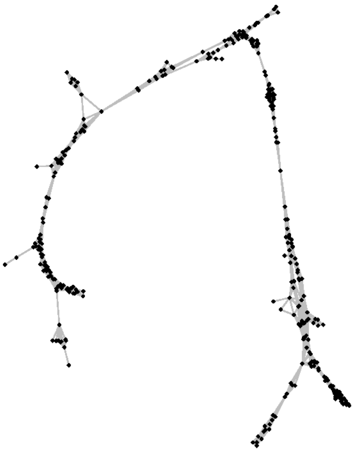

CL104

Ty3−gypsy/Tekay
Number of reads:317 (0.047%)
Number of pairs: 1728
Density: 0.0345
Mean edge weigth: 107410
Max. degree: 22

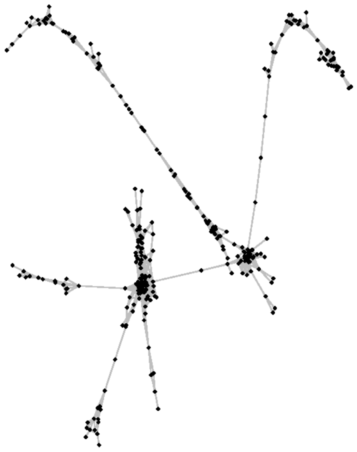

CL105

DNA transp/Mutator
Number of reads:310 (0.046%)
Number of pairs: 1841
Density: 0.03844
Mean edge weigth: 82717
Max. degree: 48

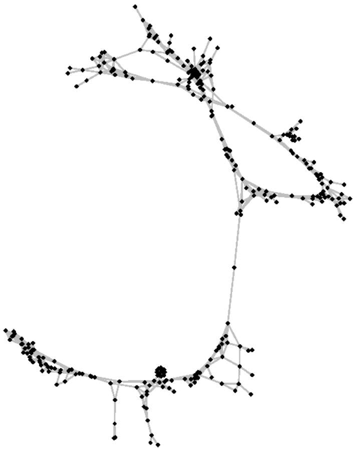

CL106

Ty3−gypsy/Athila
Number of reads:307 (0.046%)
Number of pairs: 930
Density: 0.0198
Mean edge weigth: 86413
Max. degree: 17

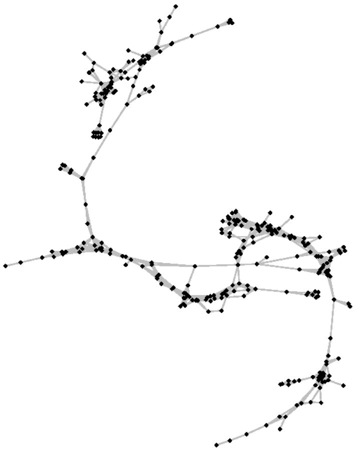

CL107

unclassified
Number of reads:299 (0.044%)
Number of pairs: 1030
Density: 0.02312
Mean edge weigth: 104110
Max. degree: 19

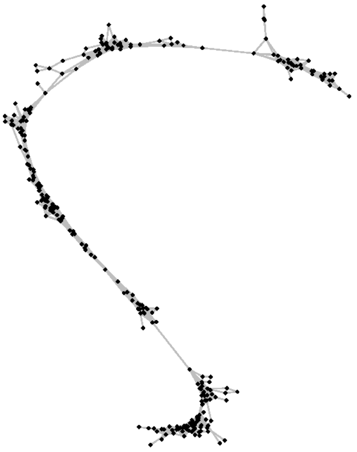

CL108

unclassified
Number of reads:279 (0.041%)
Number of pairs: 1540
Density: 0.03971
Mean edge weigth: 88794
Max. degree: 31

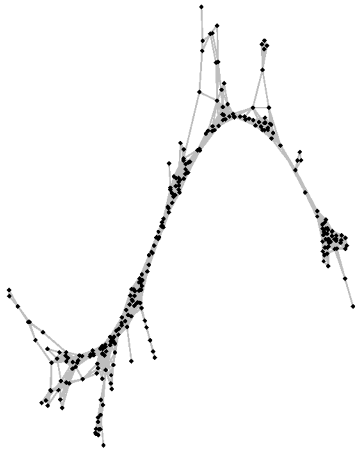

CL109

Ty3–gypsy/Athila
Number of reads:274 (0.041%)
Number of pairs: 1800
Density: 0.04813
Mean edge weigth: 81047
Max. degree: 29

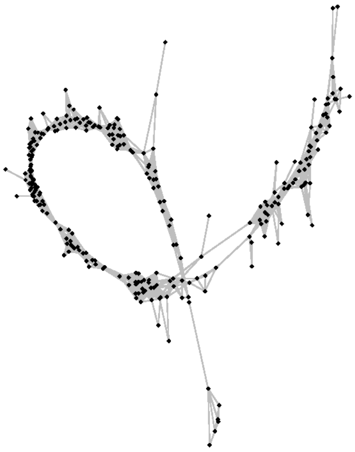

CL110

DNA transp/Mutator
Number of reads:270 (0.04%)
Number of pairs: 1679
Density: 0.04623
Mean edge weigth: 100930
Max. degree: 29

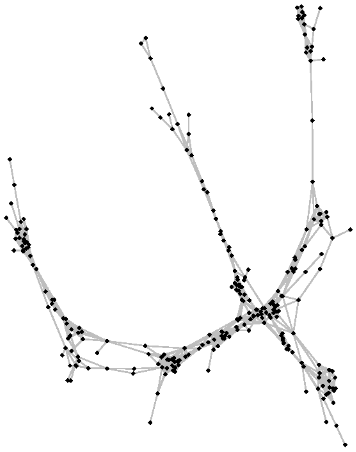

CL111

unclassified
Number of reads:261 (0.039%)
Number of pairs: 1021
Density: 0.03009
Mean edge weigth: 99400
Max. degree: 23

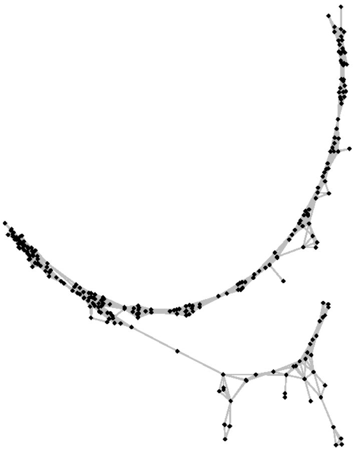

CL112

DNA transp/Mutator
Number of reads:261 (0.039%)
Number of pairs: 1315
Density: 0.03876
Mean edge weigth: 97764
Max. degree: 20

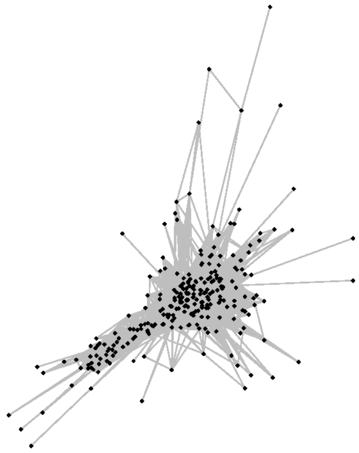

CL113

TRIM
Number of reads:252 (0.037%)
Number of pairs: 5835
Density: 0.1845
Mean edge weigth: 80827
Max. degree: 129

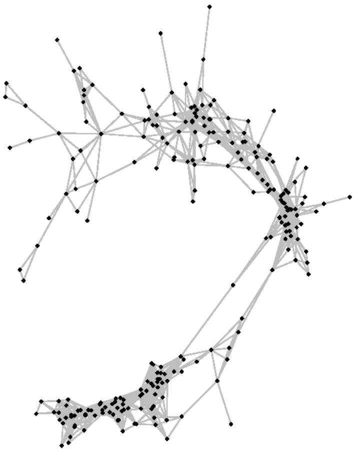

CL114

unclassified
Number of reads:237 (0.035%)
Number of pairs: 1192
Density: 0.04262
Mean edge weigth: 85770
Max. degree: 32

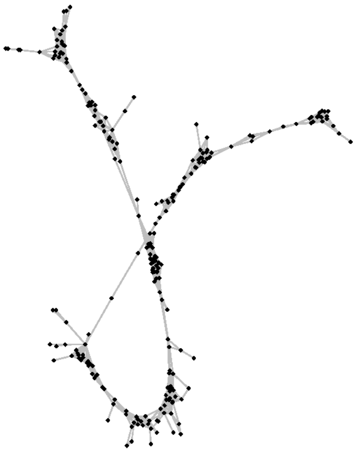

CL115

Ty3–gypsy/Athila
Number of reads:235 (0.035%)
Number of pairs: 1154
Density: 0.04197
Mean edge weigth: 87672
Max. degree: 29

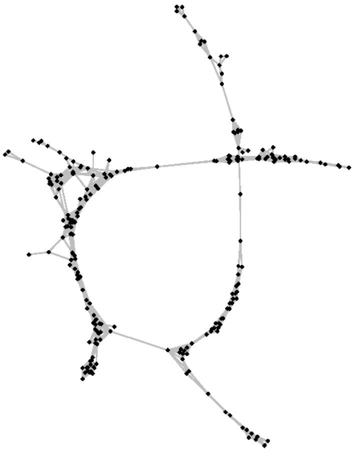

CL116

Ty1–copia/Maximus–SIRE
Number of reads:234 (0.035%)
Number of pairs: 1168
Density: 0.04285
Mean edge weigth: 108200
Max. degree: 22

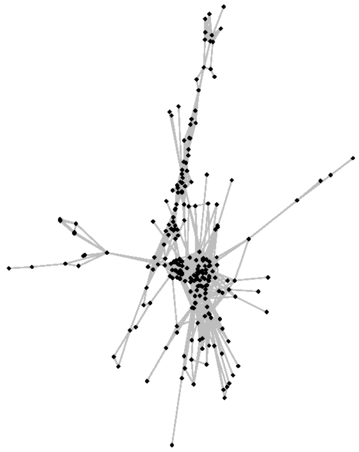

CL117

unclassified
Number of reads:228 (0.034%)
Number of pairs: 1328
Density: 0.05132
Mean edge weigth: 96675
Max. degree: 68

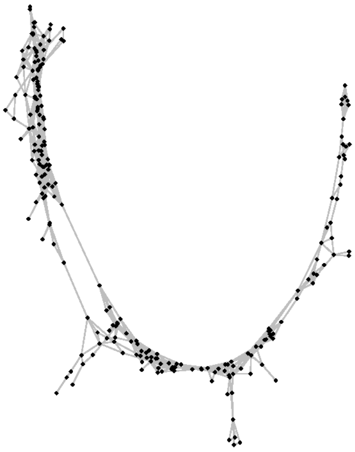

CL118

Ty1–copia/Angela
Number of reads:228 (0.034%)
Number of pairs: 790
Density: 0.03053
Mean edge weigth: 73693
Max. degree: 23

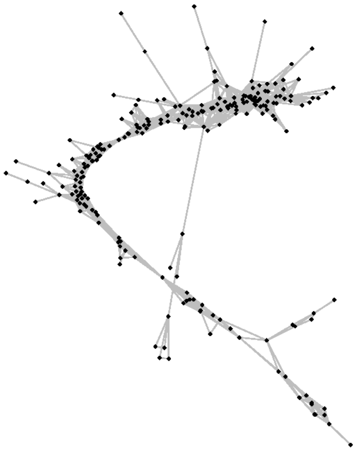

CL119

LINE
Number of reads:226 (0.034%)
Number of pairs: 1507
Density: 0.05927
Mean edge weigth: 95684
Max. degree: 41

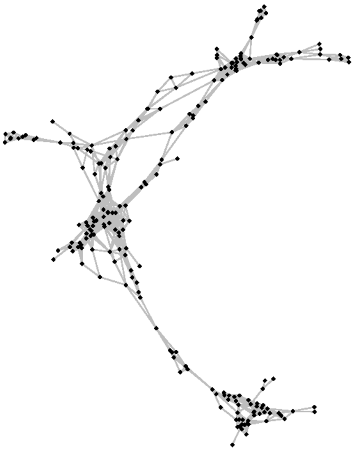

CL120

unclassified
Number of reads:222 (0.033%)
Number of pairs: 1021
Density: 0.04162
Mean edge weigth: 86117
Max. degree: 27
