# Supplementary material for: Next Generation Sequencing-Based Analysis of Repetitive DNA in the Model Dioceous Plant Silene latifolia
Source: PLoS One. 2011 Nov 9;6(11):e27335. doi: 10.1371/journal.pone.0027335 (PMC3212565; doi:10.1371/journal.pone.0027335)
Supplement: Figure S2 — Phylogenetic analysis of S. latifolia LTR-retrotransposons based on RT sequences detected in contigs assembled from 454 data. (PDF) [file pone.0027335.s002.pdf]

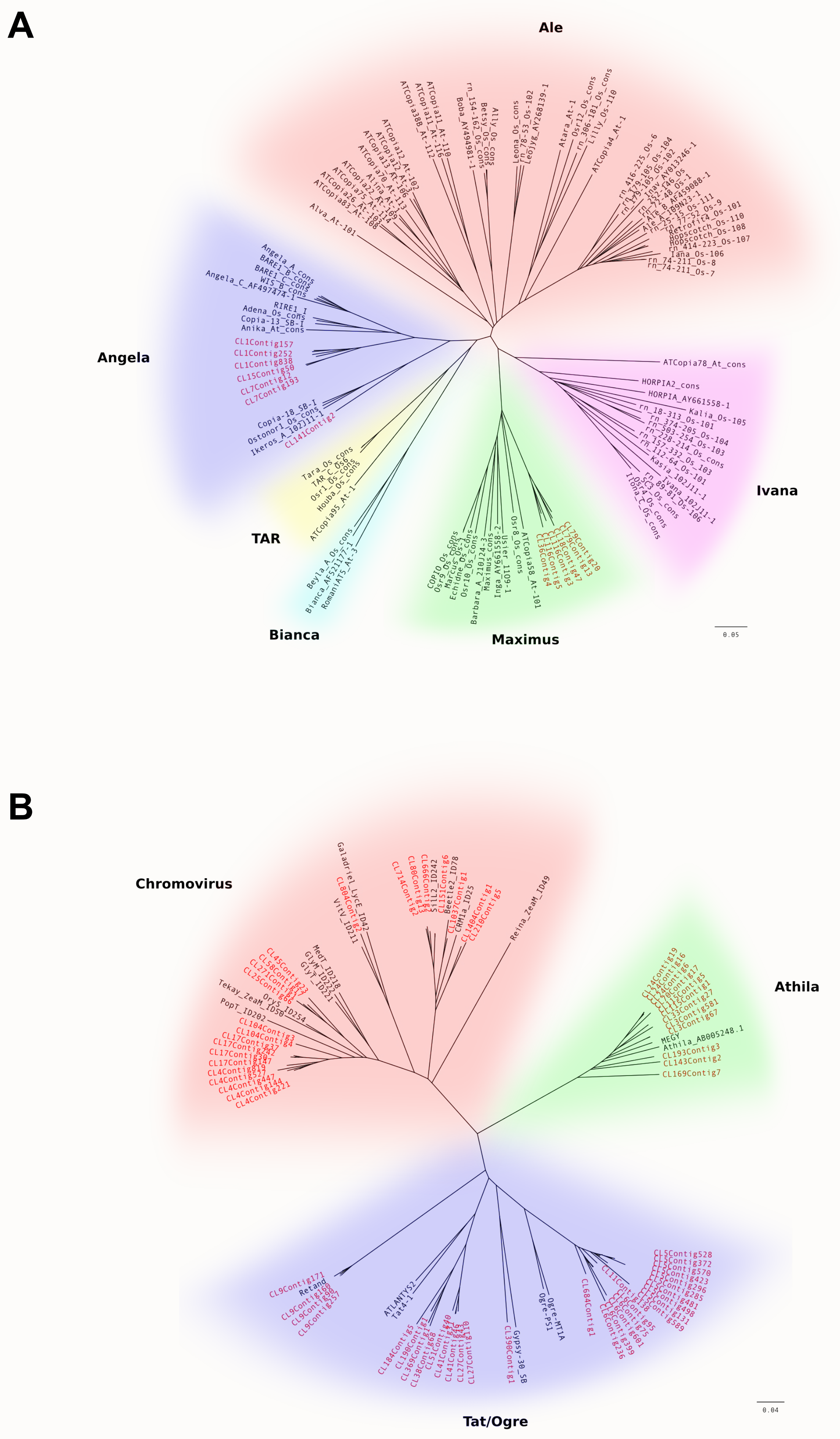

Figure S2. Phylogenetic analysis of *Silene latifolia* retrotransposons based on RT sequences. Unrooted phylogenetic trees of Ty1/copia elements (**A**) and Ty3/gypsy elements (**B**). Contigs assembled from 454 reads are highlighted and their names indicate cluster (CL) of origin.
