# Supplementary material for: Next Generation Sequencing-Based Analysis of Repetitive DNA in the Model Dioceous Plant Silene latifolia
Source: PLoS One. 2011 Nov 9;6(11):e27335. doi: 10.1371/journal.pone.0027335 (PMC3212565; doi:10.1371/journal.pone.0027335)
Supplement: Figure S3 — Analysis of rDNA sequencing bias related to GC content of template DNA. (PDF) [file pone.0027335.s003.pdf]

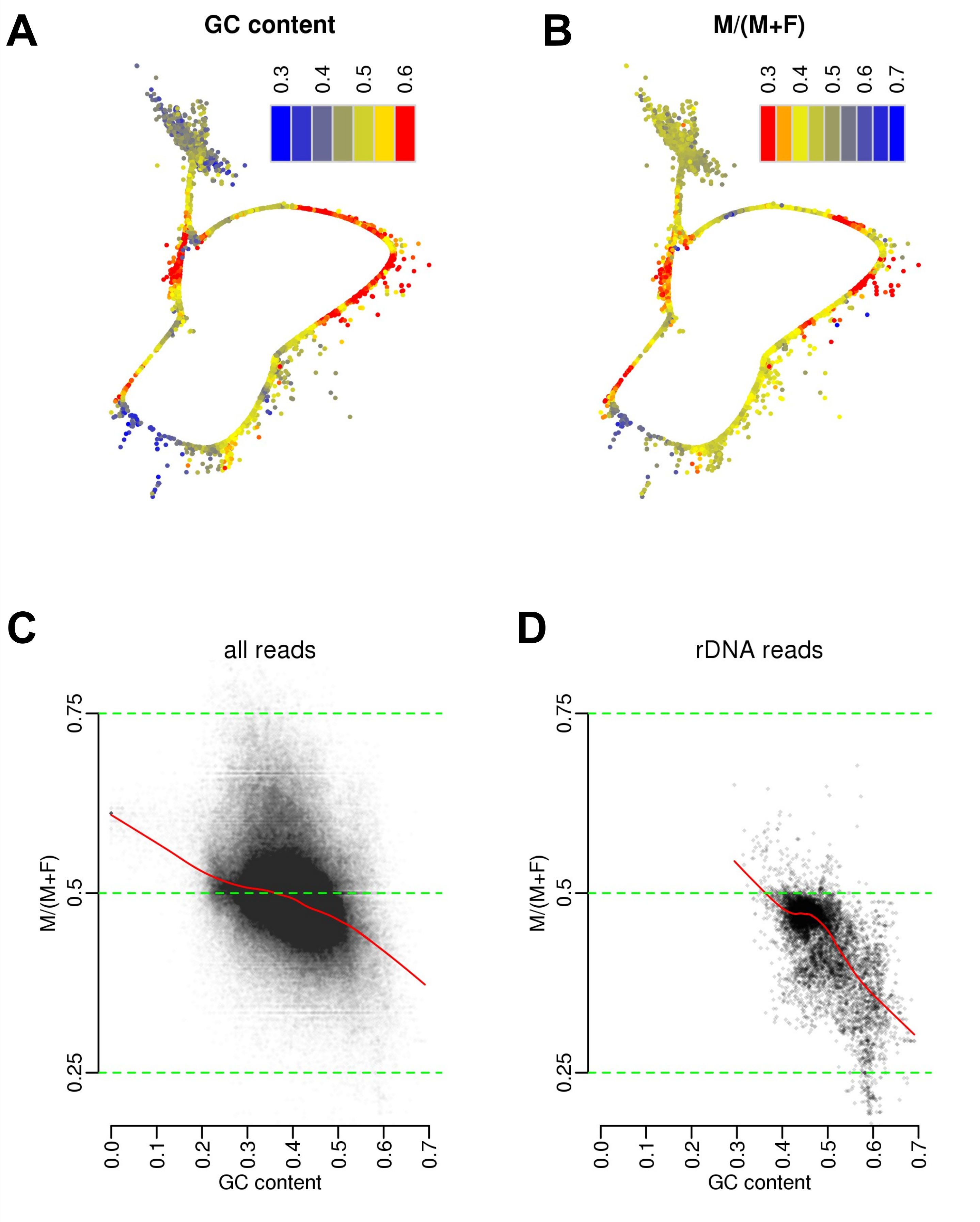


**Figure S3. Correlation of the quantification bias of rDNA repeats with their GC content.** (**A-B**) Graph layout of the rDNA repeat unit with nodes representing 454 reads (see Figure 5 for graph annotation). The reads are colored according to their GC content (**A**) or according to representation of their sequences in male (M) and female (F) Illumina reads (**B**). Values below 0.5 indicate sequence enrichment in the sample of female Illumina reads compared to the male reads. (**C-D**) Plots of sequence proportions in male and female Illumina reads in relation to the GC content of the sequence. Dots on the plots represent 454 reads and their positions along the Y-axis are determined by the number of similarity hits to Illumina reads from the male and female sample. This is shown for all *S. latifolia* 454 reads (**C**) and for rDNA reads only (**D**). The red line represents a locally weighted scatter plot smoothing fitted curve.
