# Supplementary material for: Next Generation Sequencing-Based Analysis of Repetitive DNA in the Model Dioceous Plant Silene latifolia
Source: PLoS One. 2011 Nov 9;6(11):e27335. doi: 10.1371/journal.pone.0027335 (PMC3212565; doi:10.1371/journal.pone.0027335)
Supplement: Figure S4 — Reconstruction of satellite repeats based on k -mer frequency analysis in Illumina sequencing data. (PDF) [file pone.0027335.s004.pdf]

CL16

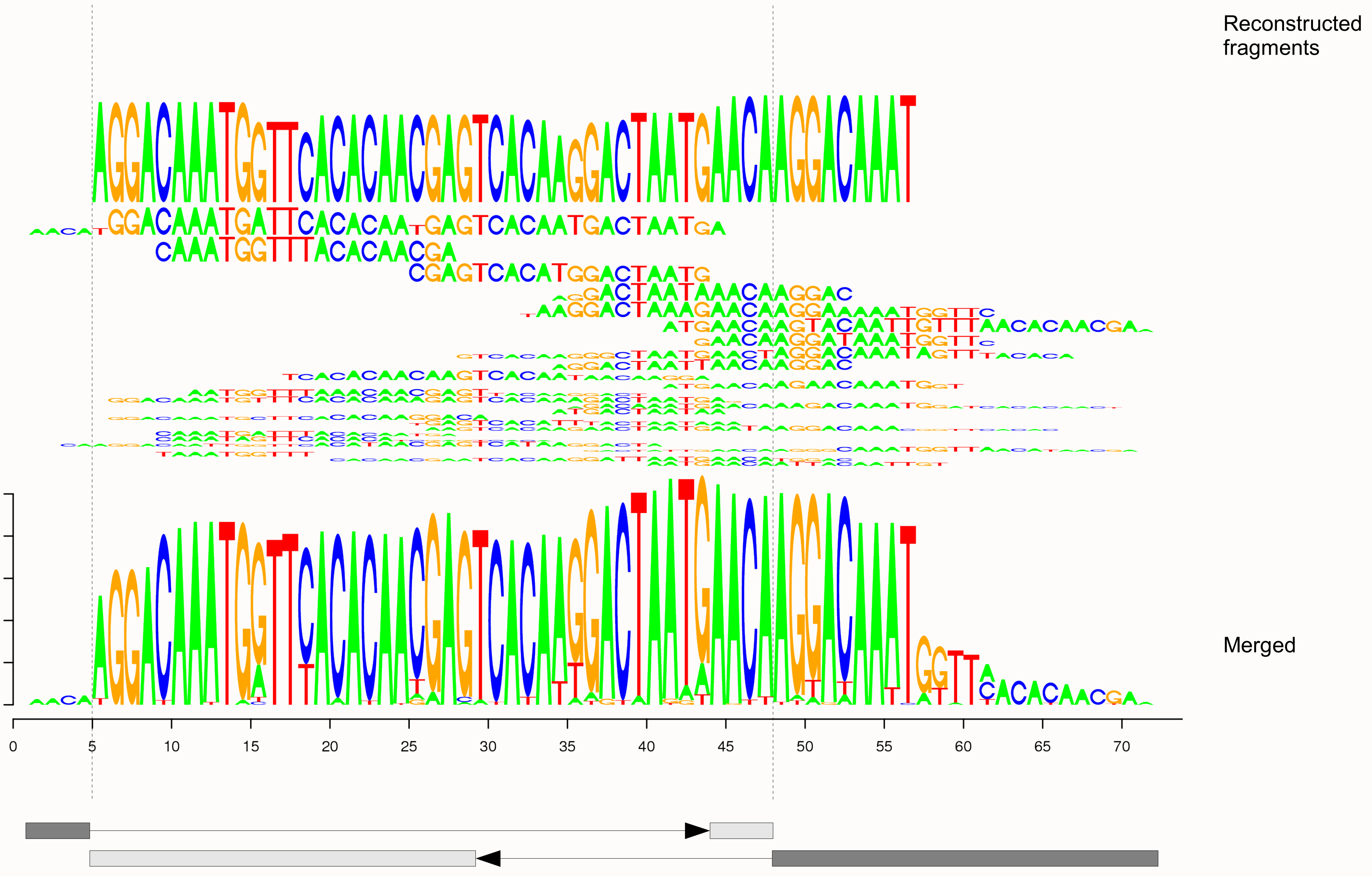

Figure S4A - Positions and sequences of reconstructed fragments used to produce CL16 consensus logo. Fragments were reconstructed from 10-mers, using a minimal seed k-mer frequency of 0.0005 and extension threshold of 20%. Vertical lines indicate the beginning and end of the monomer. To produce the final logo, sequences extending outside the monomer region were moved and merged with the monomer logo as indicated below. The same reconstruction procedure was used for the sequences displayed in panels B-E.

CL12

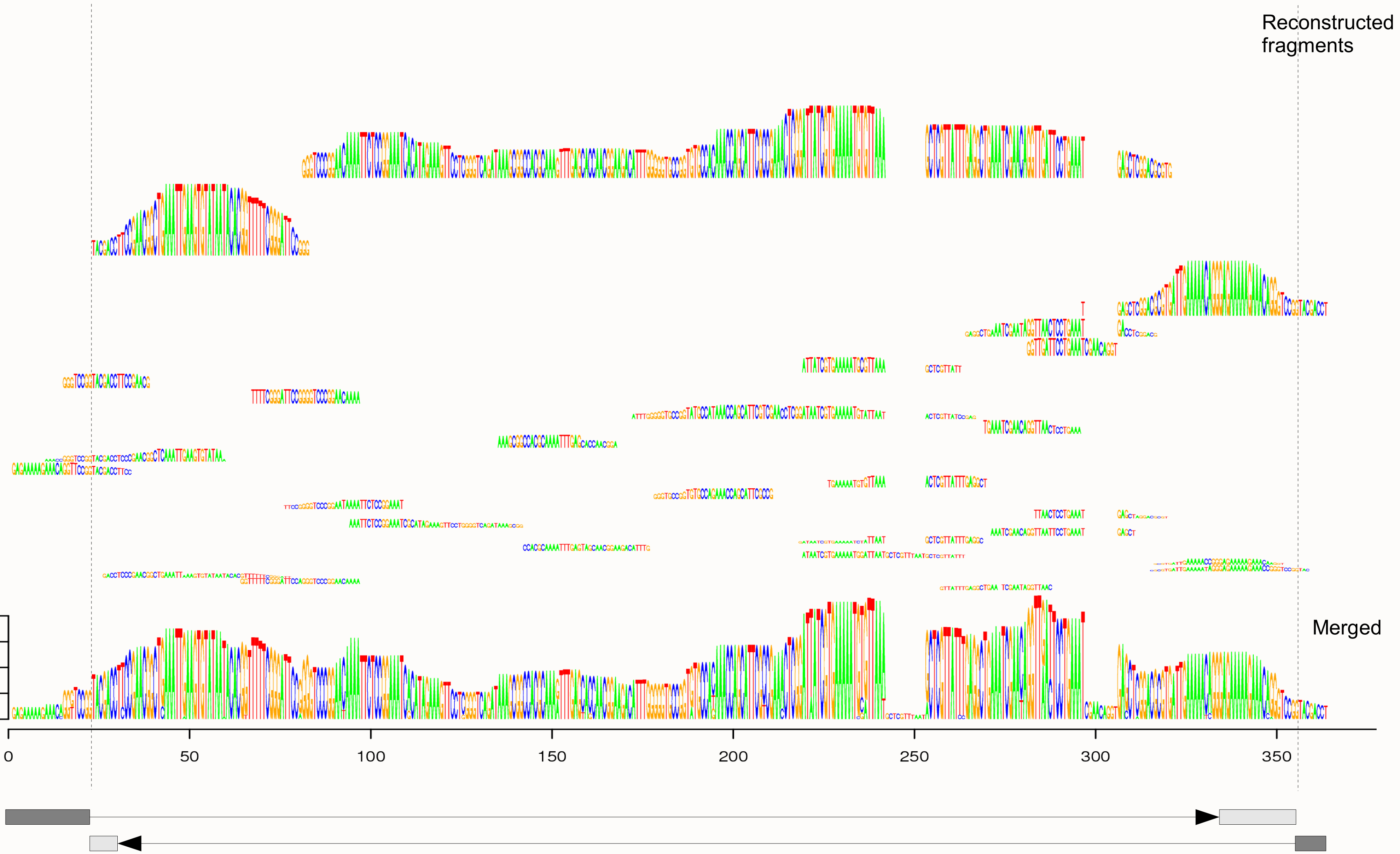

Figure S4B - Positions and sequences of reconstructed fragments used to produce CL12 consensus logo. Fragments were reconstructed from 17-mers, using a minimal seed k-mer frequency of 0.0002 and extension threshold of 20%.

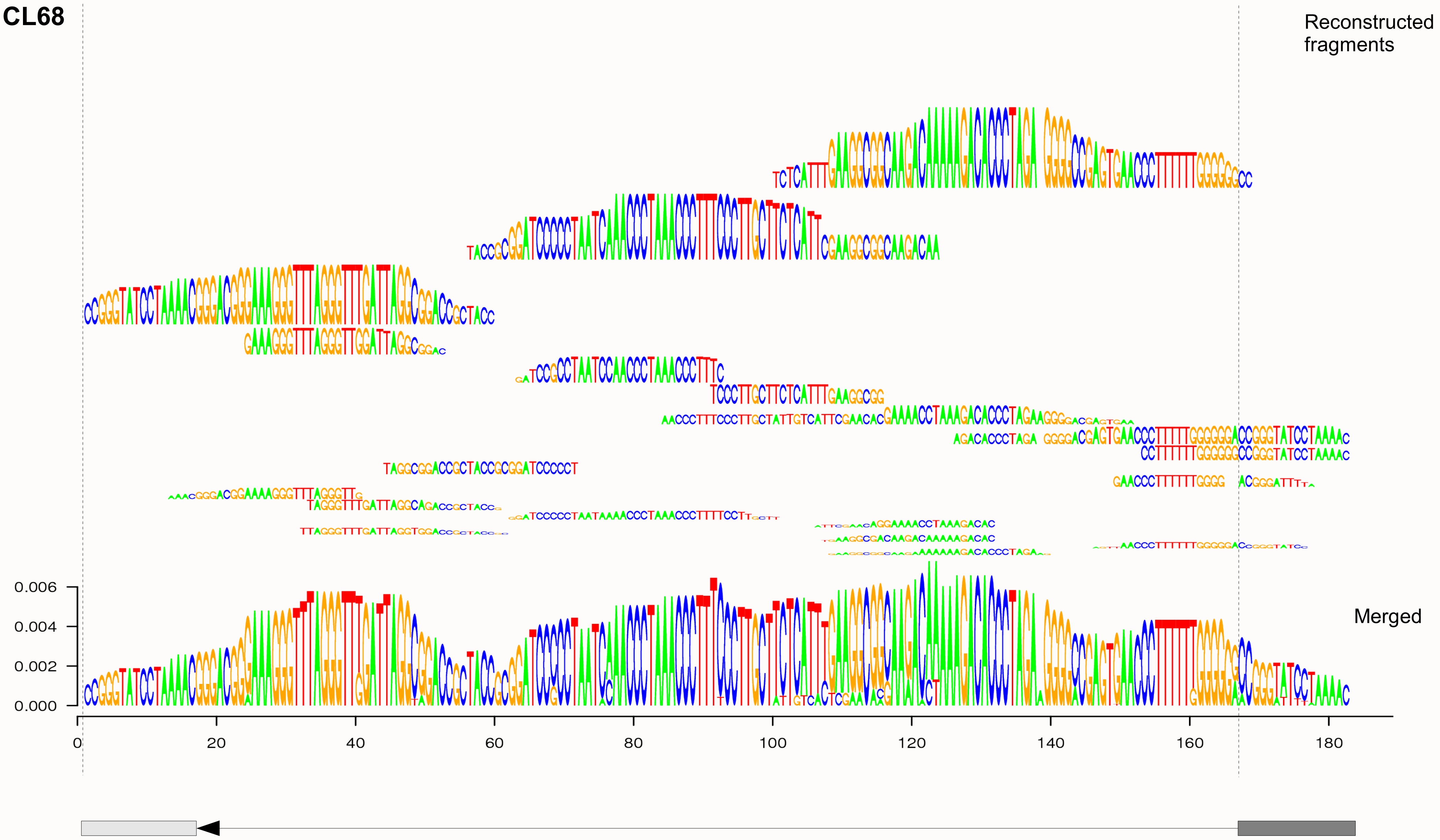

Figure S4C - Positions and sequences of reconstructed fragments used to produce CL68 consensus logo. Fragments were reconstructed from 17-mers, using a minimal seed k-mer frequency of 0.0003 and extension threshold of 20%.

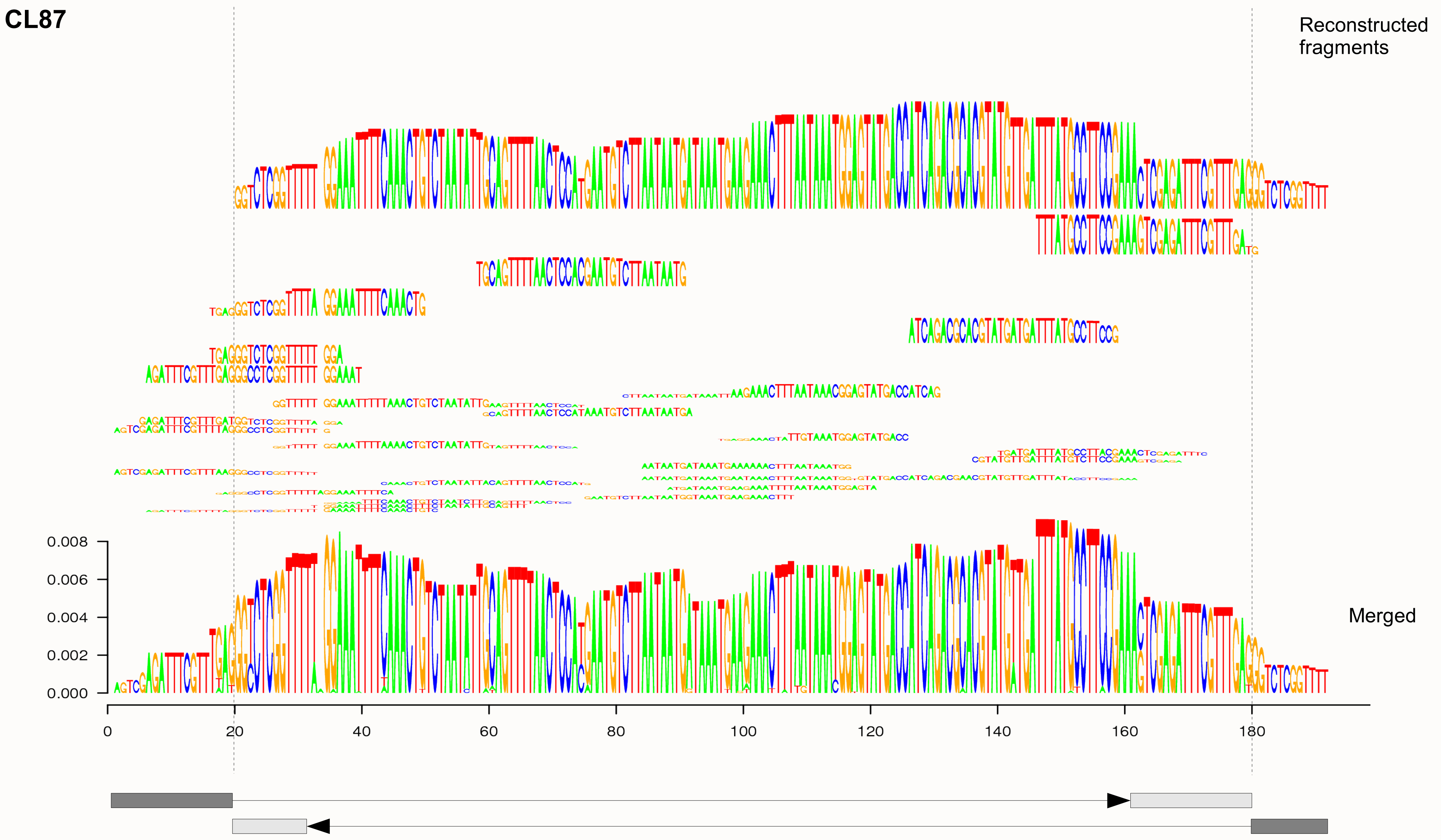

Figure S4D - Positions and sequences of reconstructed fragments used to produce CL87 consensus logo. Fragments were reconstructed from 17-mers, using a minimal seed k-mer frequency of 0.0002 and extension threshold of 20%.

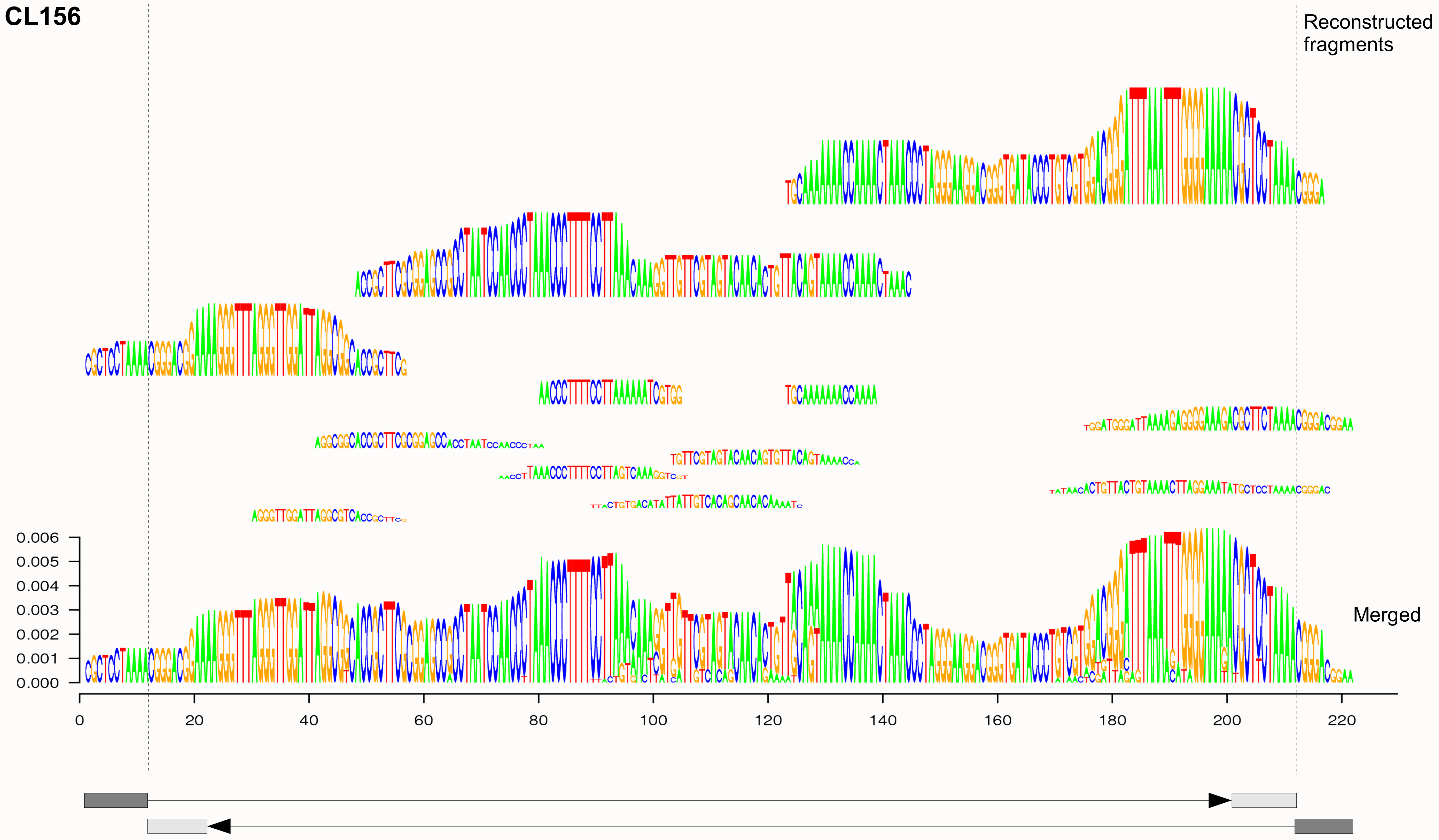

Figure S4E - Positions and sequences of reconstructed fragments used to produce CL156 consensus logo. Fragments were reconstructed from 17-mers, using a minimal seed k-mer frequency of 0.0005 and extension threshold of 20%.
